# Properties of Layered Cu-Based Composites Prepared by Spark Plasma Sintering

**DOI:** 10.3390/ma19214601

**Published:** 2026-10-30

**Authors:** Anhui Cai, Yalin Hu, Yun Luo, Qi An

**Affiliations:** 1 College of Mechanical Engineering, Hunan Institute of Science and Technology, Yueyang 414000, China; 2 School of Artificial Intelligence, Hunan Institute of Science and Technology, Yueyang 414000, China

**Keywords:** Cu_70_Zr_15_Ti_15_@Cu composite, sintering temperature, spark plasma sintering, properties

## Abstract

Bulk lamellar-structured Cu_70_Zr_15_Ti_15_@Cu composites were successfully fabricated via spark plasma sintering (SPS) of flake-like Cu_70_Zr_15_Ti_15_@Cu amorphous composite powders. These core-shell powders were synthesized by performing electroless copper plating on ball-milled flake-like Cu_70_Zr_15_Ti_15_ amorphous precursor powders. The systematic influence of sintering temperature on the microstructure evolution, hardness, surface wettability, and corrosion resistance of the consolidated composites was thoroughly investigated. The results demonstrate that the phase constitution of the Cu_70_Zr_15_Ti_15_ powders undergoes a distinct transformation: the initial Cu_51_Zr_14_ single phase transforms into Cu_51_Zr_14_ + amorphous dual-phase mixture after ball milling. The average crystallite size of the Cu matrix increases exponentially with the elevation of sintering temperature. Both the bulk density and Vickers hardness of the composites show a near-linear increasing trend as the sintering temperature rises. Meanwhile, the characteristic lamellar microstructure of the material evolves from a coarse thick-layered morphology to a refined fine–lamellar architecture with increasing sintering temperature. The electrochemical corrosion resistance of the composites in 3.5 wt% NaCl solution is progressively enhanced at higher sintering temperatures. Notably, the surface wettability of both the mechanically polished and electrochemically corroded samples exhibits a clear transition from hydrophilicity to hydrophobicity as the sintering temperature increases. Furthermore, the underlying mechanisms governing the corrosion behavior evolution and surface wettability transition are systematically elucidated.

## 1. Introduction

Cu and Cu-based alloys are widely used in electronics and electrical engineering, 5G communication, new energy vehicles, rail transit, aerospace, and naval marine engineering, owing to their exceptional combination of properties: high electrical and thermal conductivity, excellent seawater corrosion resistance, and the ability to inhibit the adhesion and growth of marine organisms [1,2,3,4]. As modern industrial technology advances rapidly, increasingly stringent service conditions impose higher performance requirements on copper alloys. For instance, the deployment of large-tonnage, high-power marine vessels demands copper alloys that can withstand harsher deep-sea environments. High-speed railway contact wires require an integrated set of properties: high tensile strength, good electrical conductivity, superior high-temperature oxidation resistance, excellent wear and corrosion resistance, and strong fatigue vibration resistance. The ongoing miniaturization and high-density integration of communication and electronic equipment also place greater demands on the strength, electrical conductivity, and thermal conductivity of copper alloys. In this context, the development of new-generation high-performance copper alloys has become an urgent industrial necessity.

High-strength and high-conductivity copper alloys refer to a class of advanced copper-based materials that simultaneously achieve a tensile strength of no less than 600 MPa and an electrical conductivity of no less than 80% IACS. To date, the main commercially applied high-performance copper alloy systems are Cu-Fe-P, Cu-Ni-Si, and Cu-Cr-Zr. Among these, Cu-Cr-Zr alloys have attracted extensive research attention due to their outstanding strength-conductivity synergy [2,3,4,5,6,7], and they have been widely adopted in applications such as integrated circuit lead frames, rail transit contact wires, high-voltage electrical contact pads, electric brush materials, end rings for high-power traction motors, and combustion chamber components for large-thrust rocket engines [2,3,4]. Previous researchers have attempted to further improve the mechanical, electrical, thermal, and tribological properties of these alloys through component optimization, heat treatment design, and advanced plastic-forming processes [5,6,7]. However, it remains a major challenge to simultaneously elevate the tensile strength, electrical conductivity, and ductility of copper alloys to the target levels of 1 GPa, 80% IACS, and 15%, respectively. This indicates that the strategy of achieving ultrahigh strength, high conductivity, and good ductility synchronously within a single conventional alloy system has encountered a clear performance bottleneck. Numerous studies have demonstrated that Cu-based composites with tailored microstructures, including core-shell, layered, sandwich, and three-dimensional interconnected network architectures, represent one of the most effective approaches to break this performance tradeoff [8,9,10,11]. Additionally, surface metallization modification with a pure Cu coating has been proven to be an effective method to significantly improve the surface wettability between different phases in the composite system [9,10,11,12].

Cu-Zr and Cu-Ti alloys have been extensively investigated and widely applied as integrated circuit lead frame materials, high-speed railway contact wires, electrical contact components, and heat exchanger components in international thermonuclear experimental reactors, due to their excellent comprehensive properties including high conductivity, high strength, good tribological performance, and superior corrosion resistance [13,14,15,16,17,18,19,20,21]. For example, Han et al. [13] fabricated a Cu-0.1 wt% Zr alloy via mechanical alloying, which exhibited an electrical conductivity of 75.19% IACS and a tensile strength of 526 MPa. Kauffmann et al. [14] reported that the ultimate tensile strength of a Cu-0.3 wt% Zr alloy can be increased from 625 MPa to 635 MPa, while the electrical conductivity is simultaneously enhanced from 60% IACS to 70% IACS by annealing the homogenized and subsequently cryo-drawn sample at 350 °C for 30 min. Liang et al. [18] prepared a Cu-3Zr alloy through powder metallurgy combined with a two-stage thermomechanical treatment, and the resulting material showed a yield strength of 593 MPa, an ultimate tensile strength of 684 MPa, and an electrical conductivity of 74% IACS. Suzuki et al. [20] found that both the electrical and thermal conductivities of Cu-Ti alloys (with Ti content of 1, 2, and 3.4 wt%) monotonically increased with extended aging time at 720 K. Cheng et al. [21] systematically investigated the effect of Ti content and post-heat treatment (350–400 °C) on the tensile properties of Cu-Ti composites (Ti = 3, 6, 10 wt%) fabricated via high-pressure cold-spray additive manufacturing. Their results showed that the ultimate tensile strength remained stable in the range of 265–285 MPa, while the ductility decreased significantly as Ti content increased. The composite with 3 wt% Ti, after heat treatment at 400 °C, achieved an optimal balance between tensile strength (270 MPa) and ductility (20% elongation). Most recently, Li et al. [22] studied the influence of Zr addition on the microstructure and properties of Cu-Ti alloys and found that the introduction of Zr can significantly promote the nucleation and precipitation of nanoscale β’-Cu_4_Ti precipitates, leading to a good strength–conductivity matching. However, the electrical conductivity of the modified alloy was still below 20% IACS. This clearly indicates that the overall strength and conductivity of current Cu-Zr and Cu-Ti alloys still require further improvement to meet the increasingly demanding industrial application requirements.

Furthermore, recent efforts have been dedicated to developing high-performance copper alloys using Cu-Zr-based metallic glasses as low-temperature bonding phases [8,23,24,25]. Xie et al. [23] fabricated nearly fully dense copper-based bulk metallic glass composites by sintering a mixture of Cu_50_Zr_45_Al_5_ spherical amorphous powder and pure Cu powder via spark plasma sintering (SPS) at a temperature below 441 °C, and the obtained composite exhibited an ultrahigh strength of 1.5 GPa and an electrical conductivity of 8 × 10^4^ Ω^−1^cm^−1^. Bao et al. [24] synthesized Cu-based amorphous composites by sintering a mixture of Cu-Zr-Al amorphous powder and CuCrZr powder at 420 °C using SPS, and the measured strength, plasticity, and electrical conductivity reached 900 MPa, 10%, and 52% IACS, respectively. Huang et al. [25] also obtained nearly fully dense Cu-based bulk metallic glass composites by SPS sintering of the mixture of Cu_50_Zr_43_Al_7_@Cu core-shell amorphous composite powder and pure Cu powder at 420 °C. Their results confirmed that the mechanical properties and electrical conductivity of the composites prepared from copper-coated Cu_50_Zr_43_Al_7_ amorphous powders are significantly superior to those of composites fabricated without the copper coating. The optimized composite achieved a strength of 721 MPa, a plasticity of 13%, and an electrical conductivity of 32% IACS. All these studies consistently demonstrate that high-performance Cu-based composites with excellent comprehensive properties can be successfully obtained by rationally combining amorphous alloy phases with pure Cu or conventional copper alloy matrices.

In addition, SPS is recognized as one of the most effective fabrication methods for metal matrix composites, owing to its distinctive advantages, including low sintering temperature, extremely short holding time, and ultrahigh heating/cooling rate [26]. It has been widely reported that the high localized stress and high transient current density generated during the SPS process can promote the rupture and in situ removal of the oxide film on the powder particle surface, effectively clean the powder surface, and significantly enhance the atomic mass transport, thereby greatly accelerating the powder densification process [26,27,28].

On the other hand, lamellar-structured Cu alloys and Cu-based composites have been proven to enable the synergistic regulation of strength and ductility, breaking the traditional strength–ductility tradeoff [29,30,31,32,33,34,35,36]. For example, Zha et al. [29] fabricated a Cu-Cr-Zr alloy with heterogeneous lamellar microstructure via a combined short-time annealing and aging treatment, and the alloy exhibited an ultimate tensile strength of 714.7 MPa and an elongation of 22.4%. The electrical conductivity increased to 85.4% IACS after 10 min of short-time annealing, and it remained at 78.7% IACS after subsequent aging treatment. Zhang et al. [32] reported that a lamellar CuCrZrY alloy can achieve a tensile strength of 527 MPa, a uniform elongation of 10.66%, an electrical conductivity of 83% IACS, and a thermal conductivity of 335.5 W/(m·K). Zeng et al. [34] developed a Cu-5 wt% Ag alloy with a unique nanolaminated grain structure, which delivered a tensile yield strength of 895 MPa, an electrical conductivity of approximately 83% IACS, and excellent thermal stability up to 400 °C. Li et al. [35] prepared a heterogeneous multilayered Cu/Cu-Cr-Zr/Cu composite by alternately stacking pure Cu sheets and Cu-Cr-Zr alloy sheets, and the optimized sample exhibited a ductility of 19.7%, a yield strength of 521 MPa, and an electrical conductivity of 80.57% IACS under the optimal processing conditions.

To the best of our knowledge, no reports have been found on preparing layered copper alloys via powder metallurgy using copper-based amorphous alloy precursor and its supercooled liquid region properties. Recently, we successfully synthesized a series of flake-like Cu_100-x_Zr_x_Ti_x_ (x = 5, 10, 15 at.%) amorphous composite powders via high-energy ball milling and systematically investigated their non-isothermal and isothermal crystallization behavior [37]. The results showed that the Cu_70_Zr_15_Ti_15_ amorphous composite powder possesses the highest amorphous phase content, as well as the lowest glass transition temperature (*T*_g_) and crystallization temperature (*T*_x_), making it particularly suitable for the low-temperature preparation of high-performance Cu-based bulk alloys. Thus, flake-like Cu_70_Zr_15_Ti_15_ amorphous composite powders were first prepared by ball milling, which provides a fundamental structural basis for the subsequent construction of lamellar-structured Cu alloys. Subsequently, the flake-like Cu_70_Zr_15_Ti_15_@Cu core-shell powders were synthesized through a controlled electroless copper plating process to further improve the interfacial bonding and electrical conductivity of the final material. Finally, bulk lamellar Cu_70_Zr_15_Ti_15_@Cu alloys were consolidated by SPS of the as-prepared flake-like Cu_70_Zr_15_Ti_15_@Cu amorphous composite powders. The microstructure evolution, phase transformation behavior, mechanical hardness, corrosion resistance, and surface wettability of the as-sintered bulk alloys were systematically characterized and analyzed.

## 2. Experimental

### 2.1. Preparation of Alloy Powders

Cu_70_Zr_15_Ti_15_ (at.%) master alloy ingots were fabricated via arc melting of high-purity elemental Cu, Zr, and Ti in a non-consumable vacuum arc furnace under a Ti-gettered high-purity argon atmosphere. The purity of all three raw metallic elements was 99.99 wt%. The furnace chamber was first evacuated to a base vacuum of 10^−5^ Pa using a combined mechanical pump and molecular pump system and then backfilled with high-purity argon (99.99 wt%) to serve as the protective melting atmosphere. To guarantee the homogeneous distribution of alloying elements, each ingot was remelted at least 5 times. After solidification and cooling, the obtained ingots were mechanically crushed into coarse pre-alloyed powders using a self-designed jaw crusher.

The as-crushed alloy powders were subsequently subjected to high-energy ball milling for 22 h in a QM-3SP2 planetary ball mill (Nanjing Nanda Instrument Co., Ltd., Nanjing, China), with a fixed ball-to-powder weight ratio of 35:1 and a constant rotational speed of 400 r/min. A complete set of agate milling jars and agate grinding balls with three different diameters (20 mm, 10 mm, and 5 mm) were used for the milling process. The milling program was set to a bidirectional intermittent operation mode: the forward and reverse rotation durations were each maintained for 1 h, with a 0.5 h idle interval between direction switches to avoid excessive temperature rise inside the milling jar. It is worth noting that the entire milling jar was fully filled with anhydrous ethanol as the process control agent to effectively prevent the fine alloy powders from oxidation and minimize contamination introduced by abrasive wear. The final as-milled flake-like powders were collected and then vacuum-sealed and stored in an inert atmosphere to avoid surface degradation before subsequent processing.

### 2.2. Synthesis of Cu_70_Zr_15_Ti_15_@Cu Composite Powders

The as-milled Cu_70_Zr_15_Ti_15_ alloy powders were first ultrasonically cleaned in 1 mol/L dilute hydrochloric acid solution for 5 min. This pre-treatment step is designed to remove residual surface oxide impurities and effectively activate the powder surface, creating a uniformly catalytic surface state that ensures the subsequent electroless copper plating proceeds smoothly. After the acid activation process, the treated alloy powders were rinsed with deionized water no fewer than 6 times to completely remove residual HCl solution and then fully transferred into a self-formulated electroless plating bath. The electroless copper plating reaction on the surface of the alloy powders was carried out for 2 h in an SHJ-4A magnetic stirring thermostatic water bath (Changzhou Guoyu Instrument Manufacturing Co., Ltd., Changzhou, China), under a constant temperature of 50 °C and a fixed pH value maintained in the range of 12–13. The 400 mL electroless plating bath was composed of 10 g EDTA-2Na, 6 g potassium sodium tartrate (C_4_O_6_H_4_KNa·4H_2_O), 0.04 g potassium ferrocyanide (K_4_Fe(CN)_6_), 0.04 g 2,2′-bipyridine, and 10 g CuSO_4_·5H_2_O, with deionized water as the solvent. After the plating reaction was completed, the obtained Cu_70_Zr_15_Ti_15_@Cu core-shell composite powders were thoroughly rinsed with deionized water to remove residual plating solution and then dried in a DZF-6020 vacuum drying oven (Shanghai Yiheng Scientific Instrument Co., Ltd., Shanghai, China) at a constant temperature of 50 °C. The final composite powders were collected after drying and then vacuum-sealed and stored in an inert atmosphere to avoid surface oxidation before subsequent spark plasma sintering processing.

### 2.3. Preparation of Bulk Cu_70_Zr_15_Ti_15_@Cu Composites

Cylindrical bulk Cu_70_Zr_15_Ti_15_@Cu composites with a dimension of Φ10 × 10 mm were consolidated using a LABOXTM-110 spark plasma sintering system(SINTER LAND Incorporation, Ltd., Tokyo, Japan) at four different sintering temperatures: 450 K, 510 K, 580 K, and 660 K. The complete sintering procedure is described as follows. A fixed mass of the as-prepared core-shell composite powders was loaded into a Φ10 × 10 mm cylindrical graphite die. The die wall was then gently tapped several times with a rubber mallet to achieve preliminary uniform powder packing and minimize uneven density distribution in the green compact. Subsequently, the graphite die assembly was transferred into the furnace chamber of the SPS system, and the chamber was evacuated to a base vacuum level below 20 Pa. The samples were heated to the pre-set target temperature at a constant heating rate of 100 K/min, held isothermally for 10 min under a uniaxial compressive load of 7 kN, and then naturally furnace-cooled down to room temperature without applied pressure. The sintered bulk samples were retrieved after full cooling and immediately vacuum-sealed and stored in an inert atmosphere to prevent surface oxidation before subsequent microstructure characterization and performance testing.

### 2.4. Structure Examination

To accurately determine the reasonable sintering temperature window, the characteristic thermal parameters, including the glass transition temperature T_g_ and the onset crystallization temperature T_x_ of the as-milled Cu_70_Zr_15_Ti_15_ alloy powders, were measured via differential scanning calorimetry (DSC). The test was performed on a DSC3 differential scanning calorimeter (Mettler Toledo, Greifensee, Switzerland) at a constant heating rate of 20 K/min under a flowing high-purity argon atmosphere (99.99 wt%) with a continuous flow rate of 60 mL/min.

The phase constitution of the crushed pre-alloyed powders, as-milled Cu_70_Zr_15_Ti_15_ alloy powders, Cu_70_Zr_15_Ti_15_@Cu composite powders, and as-sintered bulk Cu_70_Zr_15_Ti_15_@Cu composites was identified by a DX-2700 X-ray diffractometer (XRD) (Dandong Haoyuan Instrument Co., Ltd., Dandong, China) with monochromatic Cu-Kα radiation. The amorphous phase content was quantitatively analyzed using Jade 9 software based on the full-pattern XRD fitting method. The raw XRD pattern was preprocessed by K_α2_ stripping, instrumental background subtraction, and gentle noise smoothing. All sharp crystalline diffraction peaks were identified and indexed via the PDF standard card database and then separated from the broad amorphous halo with a full width at half maximum larger than 3° 2θ using the built-in pseudo-Voigt peak fitting function. The integral areas of all crystalline peaks and the amorphous halo were extracted after fitting convergence. The amorphous phase content was calculated as the percentage of the amorphous halo integral area relative to the sum of the crystalline peak integral area and the amorphous halo integral area. To minimize manual operation error, the peak separation and integration process was repeated three times independently, and the final reported amorphous content was the arithmetic average of the three calculated results.

The microstructure and morphological features of the as-milled alloy powders, core-shell composite powders, and sintered bulk composites were systematically characterized using a BX53 Olympus optical microscope (OM) (Olympus Corporation, Tokyo, Japan) and a SH4000M scanning electron microscope (SEM) (HIROX, Tokyo, Japan). The cross-sections of bulk samples for microstructure observation were sequentially ground with SiC sandpapers of different grit sizes, followed by final polishing with 0.25 μm diamond polishing paste to obtain a scratch-free mirror surface. The elemental distribution and composition of the samples were analyzed by the energy-dispersive X-ray spectrometry (EDS) system integrated with the SEM. X-ray photoelectron spectroscopy (XPS) measurements were carried out on the corroded sample surfaces using a Thermo Fisher Scientific (Waltham, MA, USA) K-alpha spectrometer with a monochromatic Al K_α_ X-ray source. All collected XPS spectra were processed and peak-fitted using Avantage software, with a Shirley-type background subtraction. The binding energy axis of all spectra was charge-corrected by referencing the adventitious C 1s spectral component (C-C, C-H bonds) fixed at 284.8 eV.

### 2.5. Performance Test

The bulk density of sintered Cu_70_Zr_15_Ti_15_@Cu composites was measured based on Archimedes’ principle in deionized water, using an HTY-120SL high-precision solid–liquid density analyzer (Taizhou Hengxin Instrument & Equipment Co., Ltd., Taizhou, China). To minimize measurement error, each sample was repeatedly tested for 5 independent times, and the final reported bulk density value was taken as the arithmetic mean of these five valid measurements. The Vickers microhardness was tested on an YZHV-1000C multifunctional microhardness tester (Shanghai Yizong Precision Instrument Co., Ltd., Shanghai, China). The measurement was carried out under a constant indentation load of 4.9 N with a 15 s dwell time. The final reported Vickers microhardness of each sample was the average value collected from five randomly selected independent positions on the test surface.

The electrochemical corrosion behavior of the samples was evaluated via a standard three-electrode system on an RST-5000F electrochemical workstation, with 3.5 wt% NaCl aqueous solution serving as the simulated seawater corrosion medium. The open circuit potential (OCP) test was conducted for at least 1800 s until the potential fluctuation was controlled within ±1 mV, to ensure the system reached a stable electrochemical state. The potentiodynamic polarization curve was subsequently collected at a constant potential sweep rate of 1 mV/s. The electrochemical impedance spectroscopy (EIS) test was performed over a frequency range from 0.1 Hz to 100 kHz, with the excitation AC amplitude set to 10 mV. The EIS test was carried out at the steady-state open circuit potential obtained from the previous OCP measurement. In this three-electrode configuration, a platinum plate was used as the counter electrode, and a saturated calomel electrode (SCE) was employed as the reference electrode. The mirror-polished test surface of the sample was defined as the working electrode, while the remaining part of the specimen was fully encapsulated in non-conductive thermosetting resin to ensure complete electrical isolation and expose only a fixed 1 cm^2^ test area to the electrolyte. The corroded samples were collected after repeatedly rinsing with deionized water and drying, and they were then vacuum-sealed and stored in an inert atmosphere to avoid surface contamination before subsequent XPS and SEM measurements.

The surface wettability of the bulk composites was characterized via the static sessile drop method using deionized water as the test liquid. The contact angle measurement was performed on an FCA2000A contact angle goniometer equipped with a high-resolution optical camera system (Shanghai Aifeisi Precision Instrument Co., Ltd., Shanghai, China). A 4 μL deionized water droplet was carefully deposited on the flat mirror-polished sample surface, and the static morphology of the droplet was captured immediately by the optical camera. For each selected measurement point on the surface, the contact angle value was calculated as the arithmetic average of the left and right contact angles of the droplet profile. The final reported contact angle of each sample was the average value collected from five randomly selected independent positions on the test surface.

## 3. Results

**Figure 1:**
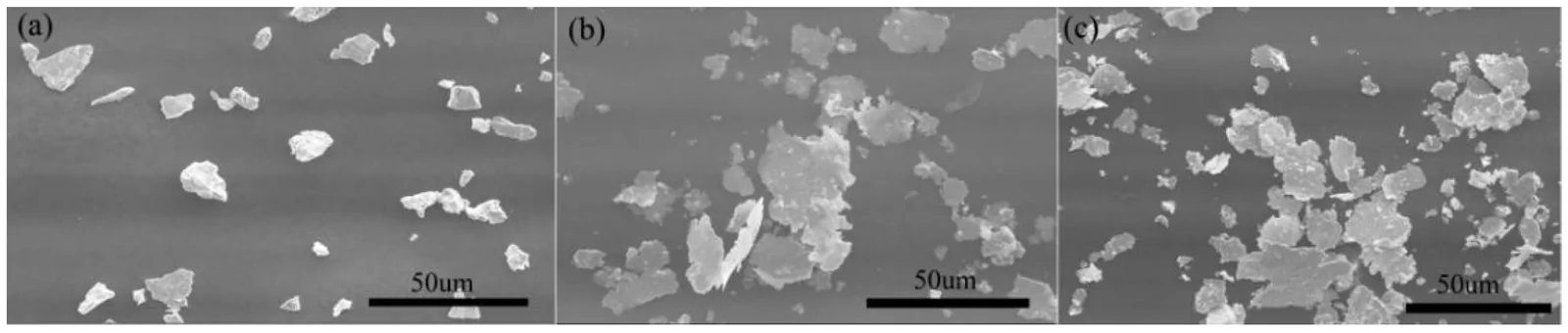
SEM morphology of the Cu_70_Zr_15_Ti_15_ powders, (**a**) as-crushed, (**b**) ball-milled and (**c**) Cu-plated.

Figure 1 presents the typical SEM morphologies of the mechanically crushed pre-alloyed powders, 22 h high-energy ball-milled Cu_70_Zr_15_Ti_15_ amorphous powders, and the final as-synthesized Cu_70_Zr_15_Ti_15_@Cu core-shell composite powders. As observed in Figure 1a, the initial crushed pre-alloyed powders exhibit a typical irregular blocky morphology with a broad particle size distribution. After 22 h of planetary high-energy ball milling, the powders were significantly flattened and transformed into a uniform flake-like microstructure, as displayed in Figure 1b. As shown in Figure 1c, the obtained Cu_70_Zr_15_Ti_15_@Cu composite powders still retain the intact flake-like morphology without obvious agglomeration, demonstrating that the pre-milled flaky powders maintain excellent dispersion state throughout the entire electroless copper plating process.

Furthermore, surface-scanning mode EDS characterization was performed on both the as-milled bare alloy powders and the final core-shell composite powders to systematically verify two key points: whether the as-milled powders introduced unexpected contamination during the long-time ball milling process, and whether elemental Cu was successfully deposited on the surface of the flaky alloy powders. The corresponding EDS quantitative results are summarized in Table 1. It can be clearly identified from the data in Table 1 that the measured Cu atomic percentage of the Cu_70_Zr_15_Ti_15_@Cu composite powders is notably higher than that of the bare as-milled powders, providing direct evidence that a copper coating was successfully deposited on the surface of the flaky alloy powders via the electroless plating reaction. The measured elemental composition of the as-milled powders shows excellent consistency with the nominal designed alloy composition of Cu_70_Zr_15_Ti_15_. Within the detection limit of EDS, no impurity elements were detected in the as-milled powders, confirming that the powders were almost free of contamination during the entire high-energy ball milling process. It is worth noting that the average thickness of the deposited copper coating is estimated to be approximately 0.3 μm based on the cross-sectional SEM observation of the composite powders (the corresponding image is not presented here).

**Figure 2:**
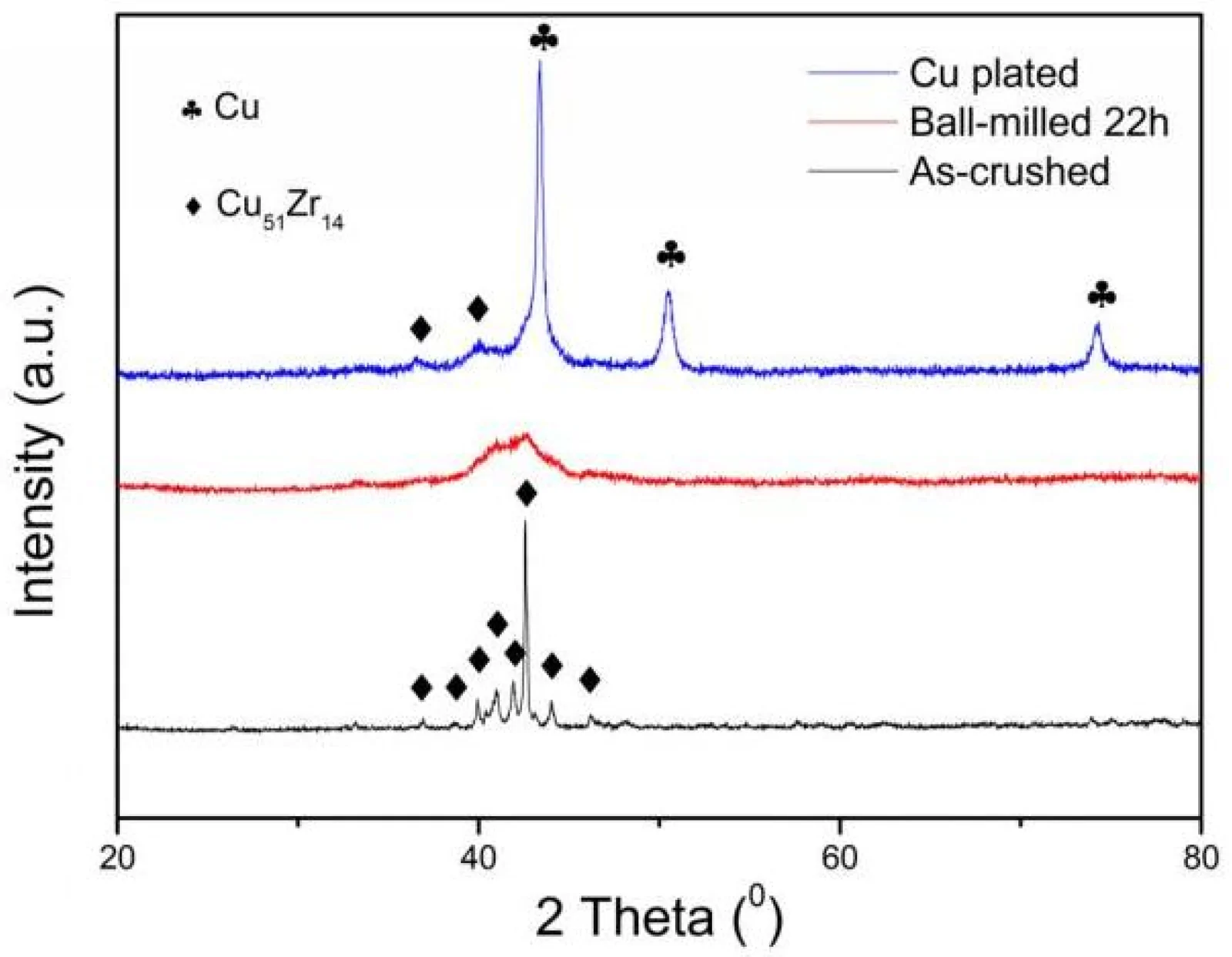
XRD patterns for as-crushed powders, ball-milled powders and Cu_70_Zr_15_Ti_15_@Cu powders.

The XRD patterns of the mechanically crushed pre-alloyed powders, 22 h high-energy ball-milled Cu_70_Zr_15_Ti_15_ powders, and as-synthesized Cu_70_Zr_15_Ti_15_@Cu core-shell composite powders are collectively presented in Figure 2. The initial crushed pre-alloyed powders exhibit a fully crystalline state, where all distinct sharp Bragg diffraction peaks can be perfectly indexed to the intermetallic phase Cu_51_Zr_14_. After 22 h of planetary high-energy ball milling, a broad diffuse amorphous halo located in the 2θ range of 38° to 45° can be clearly observed in the XRD pattern, directly demonstrating the formation of a large amount of amorphous phase in the milled powders. Meanwhile, residual weak Bragg diffraction peaks corresponding to the crystalline Cu_51_Zr_14_ phase can still be detected, indicating that a small amount of untransformed crystalline phase remains in the milled product. The flake-like morphology and partial amorphousization behavior of the 22 h milled powders in this work show high similarity to the reported results of Cu_70_Zr_15_Ti_15_ powders milled for 16 h at a rotational speed of 500 r/min with a ball-to-powder weight ratio of 40:1 [37]. In the XRD pattern of the final Cu_70_Zr_15_Ti_15_@Cu composite powders, a new set of characteristic Bragg diffraction peaks corresponding to face-centered cubic Cu phase can be clearly identified, which provides unambiguous phase-level evidence confirming that metallic copper was successfully deposited on the surface of the amorphous-matrix flaky powders via the electroless plating process.

**Figure 3:**
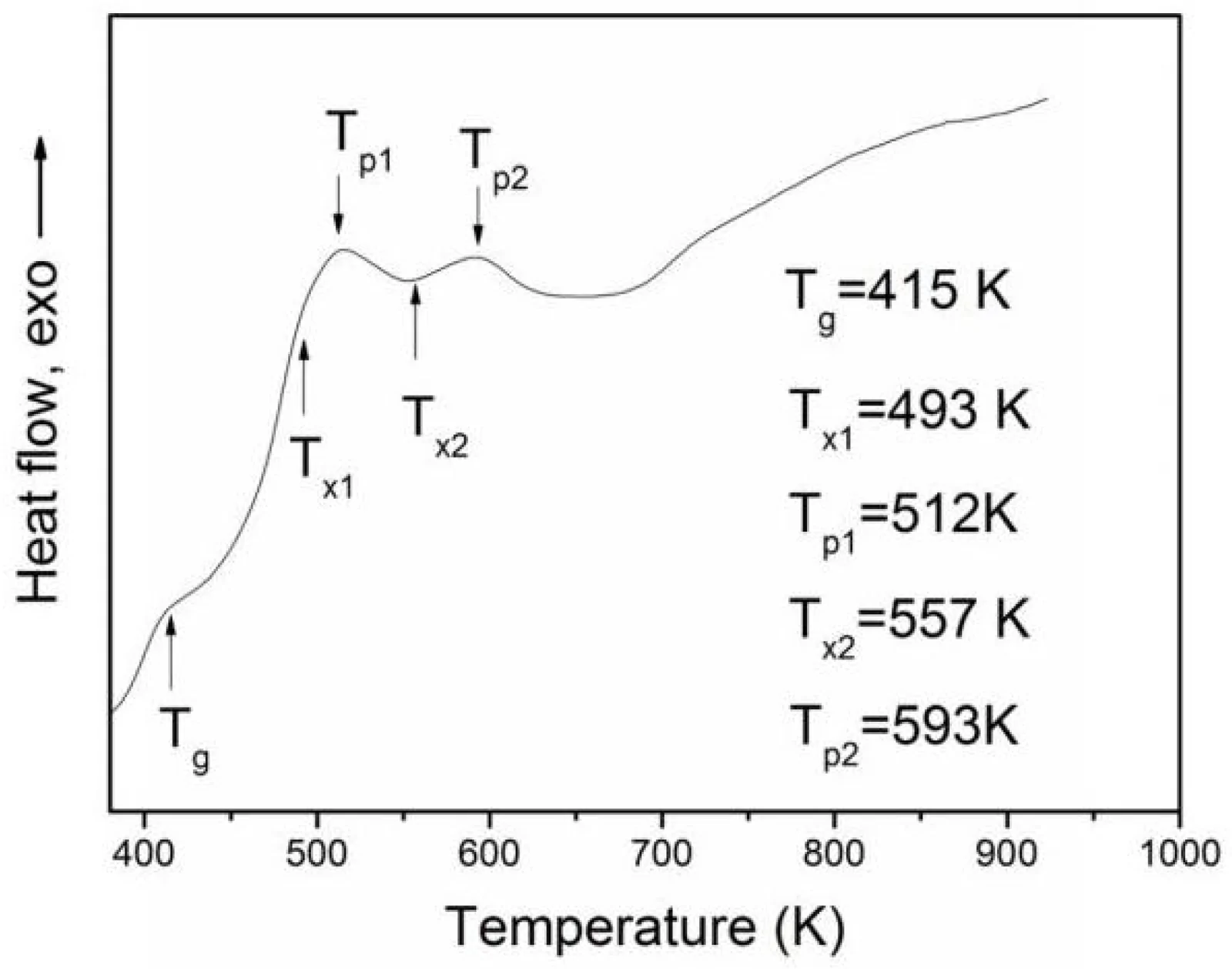
DSC curve of the as-milled Cu_70_Zr_15_Ti_15_ powders at 20 K/min heating rate.

Figure 3 presents the DSC curve of the as-milled Cu_70_Zr_15_Ti_15_ amorphous composite powders tested at a constant heating rate of 20 K/min. As clearly observed from the curve, a distinct endothermal event corresponding to the glass transition process appears in the low-temperature region, followed by two separate sequential exothermal events originating from the two-step crystallization behavior of the amorphous matrix. This thermal feature unambiguously confirms that the as-milled Cu_70_Zr_15_Ti_15_ powders are dominated by an amorphous structure, which shows excellent consistency with the broad diffuse amorphous halo observed in the XRD pattern in Figure 2. The key characteristic thermal parameters, including the glass transition temperature T_g_, the first onset crystallization temperature T_x1_, the first crystallization peak temperature T_p1_, the second onset crystallization temperature T_x2_, and the second crystallization peak temperature T_p2_, were systematically identified from the DSC curve and marked with corresponding arrows in Figure 3. The measured values of these characteristic temperatures are 415 K, 493 K, 512 K, 557 K, and 593 K, respectively. The width of the undercooled liquid region, calculated as ΔT_x_ = T_x1_ − T_g_, reaches 78 K, demonstrating that the as-milled Cu_70_Zr_15_Ti_15_ amorphous composite powders possess relatively high thermal stability. Based on these DSC results, the four sintering temperatures for consolidating the bulk Cu_70_Zr_15_Ti_15_@Cu composites were deliberately selected as 450 K, 510 K, 580 K, and 660 K, respectively.

**Figure 4:**
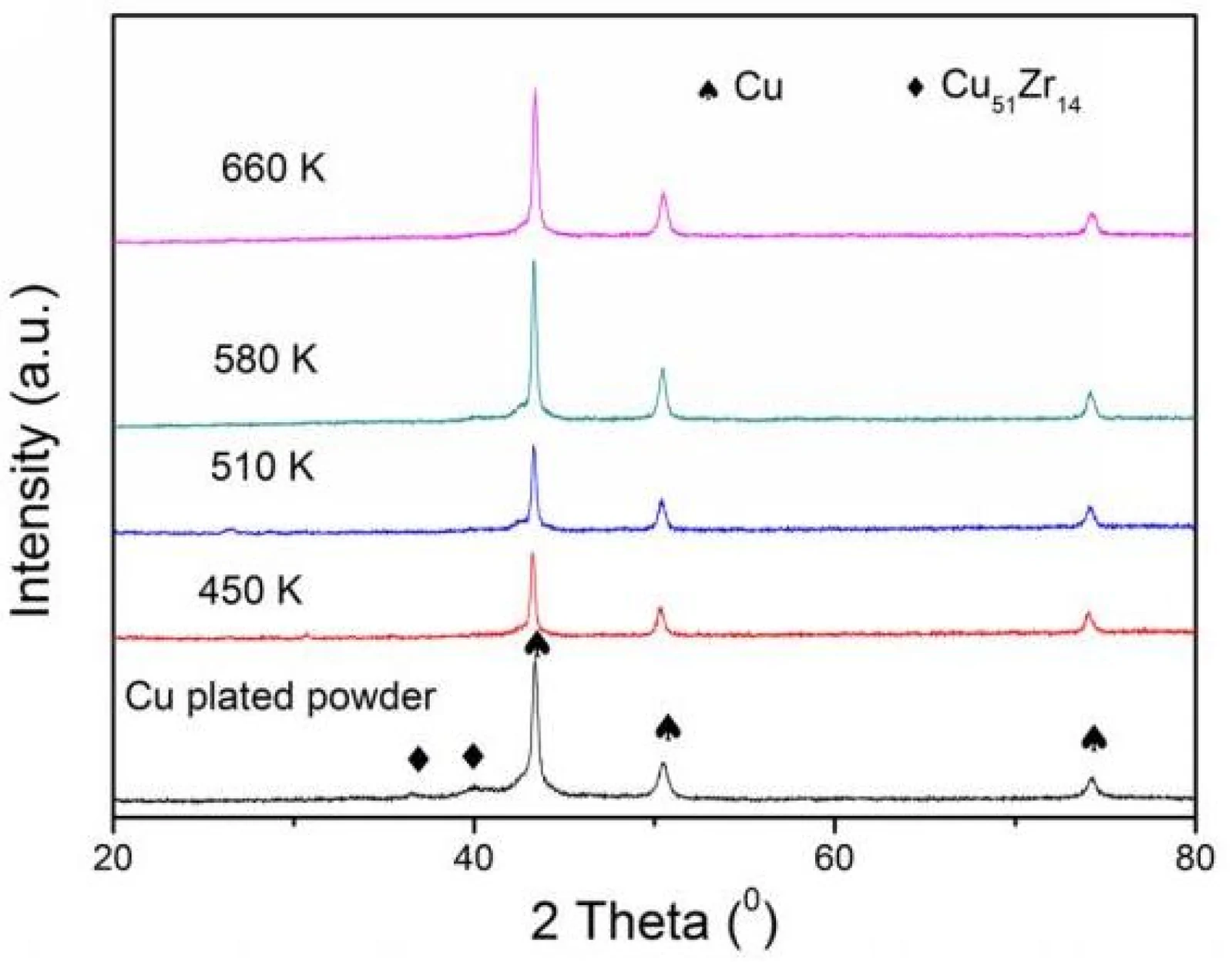
XRD patterns for Cu_70_Zr_15_Ti_15_@Cu powders and corresponding bulk samples at different sintering temperatures.

**Figure 5:**
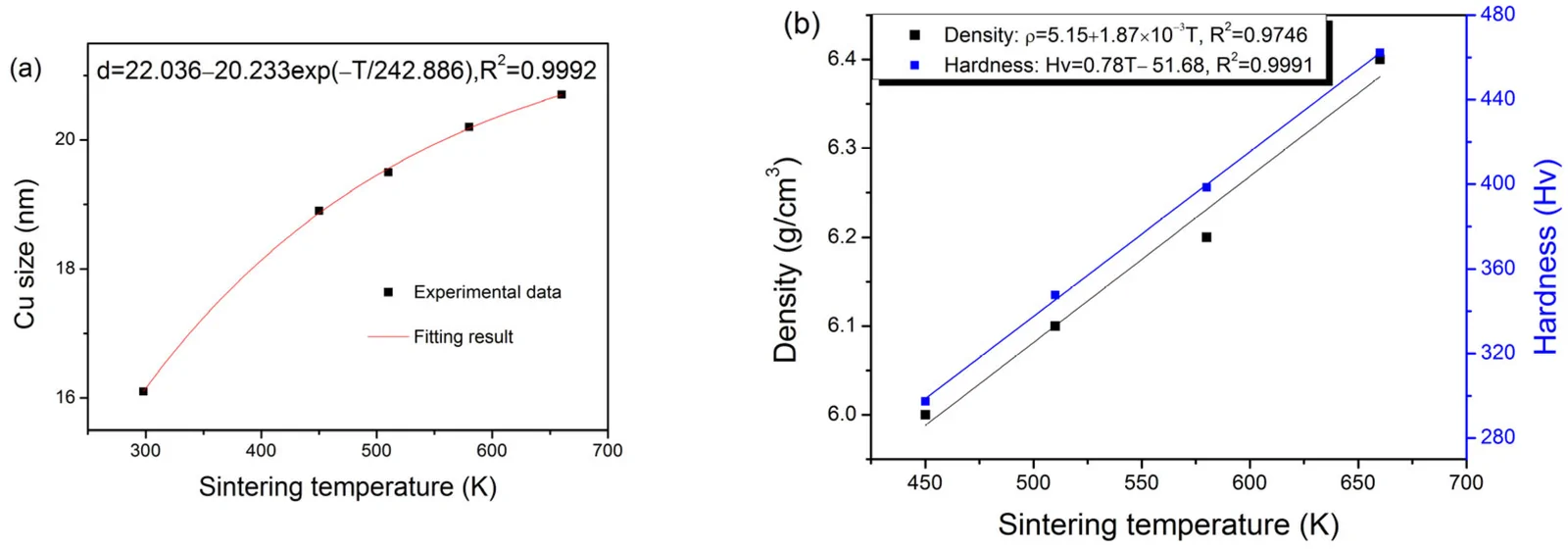
(**a**) Dependence of the Cu crystallite size on the sintering temperature. (**b**) Dependence of the density and the hardness on the sintering temperature.

The bulk Cu_70_Zr_15_Ti_15_@Cu composite samples were successfully consolidated via spark plasma sintering (SPS) under a uniaxial pressure of 7 kN for 10 min at the aforementioned sintering temperatures. Figure 4 presents the XRD patterns of the bulk composites sintered at different temperatures. It is evident that the characteristic diffraction peaks of the intermetallic Cu_51_Zr_14_ phase are no longer detectable within the sensitivity limit of XRD, suggesting that the residual crystalline phase from the milled powders has either dissolved into the matrix or transformed during the sintering process. The average crystallite size (d) of the face-centered cubic (fcc) Cu phase was calculated using the Scherrer equation—d = Kλ/(Bcos θ)—where K = 0.89 is the shape factor, λ is the wavelength of Cu-K_α_ radiation, θ is the Bragg diffraction angle, and B is the full width at half-maximum (FWHM) of the diffraction peak. The calculated values are summarized in Table 2. As shown in Figure 5a, the average Cu crystallite size increases monotonically with increasing sintering temperature. This dependence can be well described by an exponential fitting function: d = 22.036 − 20.233exp(−*T*/242.886).

Furthermore, the bulk density (ρ) and Vickers microhardness (Hv) of the bulk composites were measured and listed in Table 2. Both density and hardness exhibit a positive correlation with the sintering temperature, indicating that higher sintering temperatures promote better densification and structural compactness of the bulk samples. The theoretical density of Cu_70_Zr_15_Ti_15_ alloy is estimated as 7.5 g/cm^3^ by using the rule of mixtures. The bulk density of the SPSed samples is in the range of 6.0~6.4 g/cm^3^, indicating that the bulk samples are not fully densified. It would be due to the Cu coating inhibiting densification at low sintering temperature and short holding time. The Vickers microhardness of the prepared lamellar Cu composites ranges from 297.4 Hv to 462.2 Hv. Notably, these values are significantly higher than those reported for other copper-based alloys, such as Cu-2Ti-xZr alloys [22], lamellar Cu-Cr-Zr alloys [29], lamellar Cu-0.1 at.% Zr alloys [30], and nanolaminated Cu-5 wt.% Ag alloys processed by accumulative roll bonding [34]. Linear regression analysis reveals that the density and hardness follow the relationships *ρ* = 5.15 + 1.87 × 10^−3^*T* and Hv = 0.78*T* − 51.68, respectively, as shown in Figure 5b. This trend of increasing hardness with sintering/annealing temperature is consistent with previous studies on bulk amorphous alloy composites [38,39,40]. Additionally, the amorphous phase content of both the initial composite powders and the sintered bulk samples was quantified using Jade 9 software (Table 2). The amorphous fraction decreases progressively with increasing sintering temperature and approaches zero when the temperature reaches 580 K, indicating complete crystallization of the amorphous matrix at this stage.

**Figure 6:**
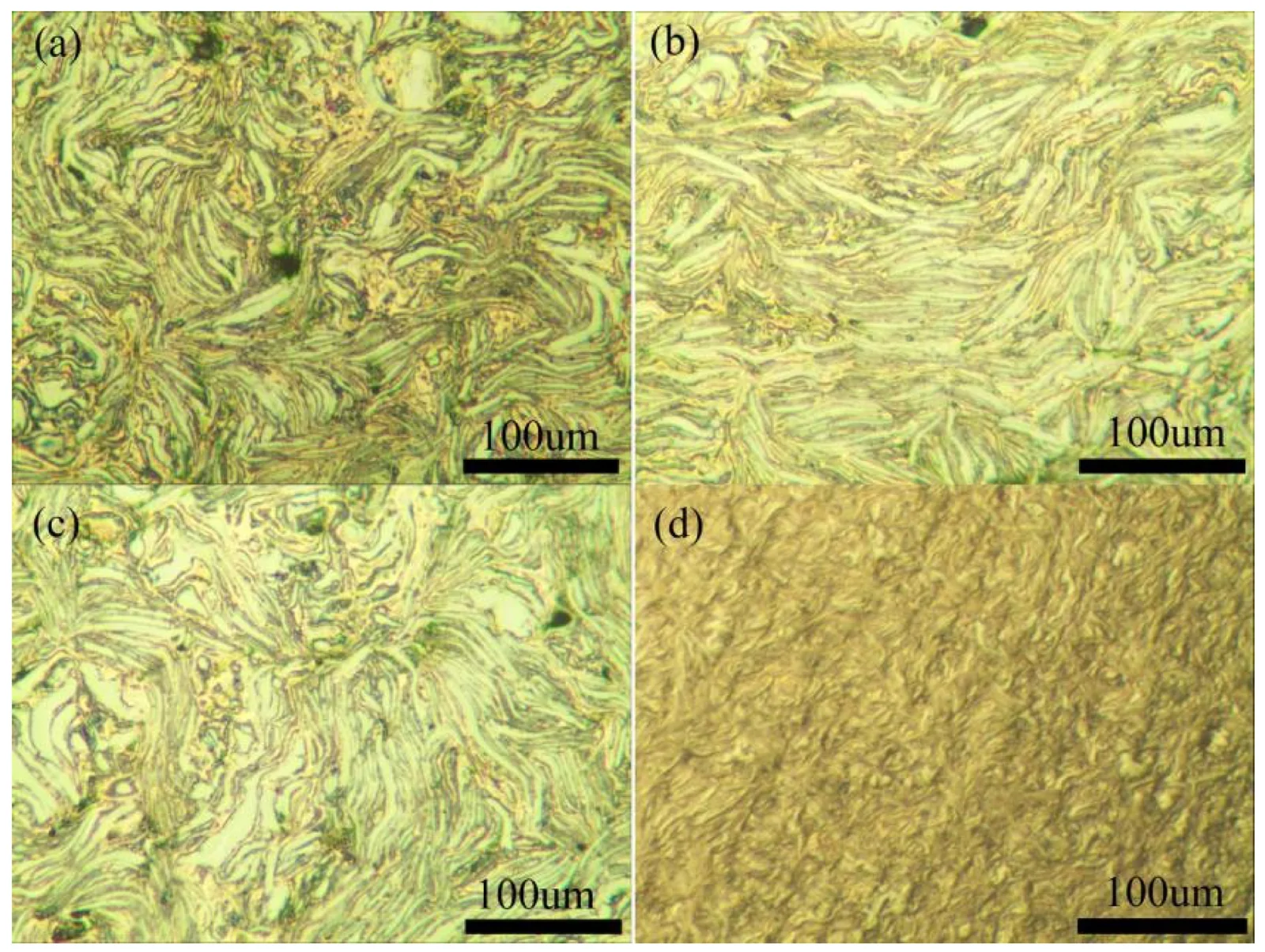
OM morphology of the samples sintered at different temperatures. (**a**) 450 K, (**b**) 510 K, (**c**) 580 K, (**d**) 660 K.

The optical microscope (OM) micrographs of the bulk Cu_70_Zr_15_Ti_15_@Cu composites sintered at different temperatures are presented in Figure 6. The microstructures of all bulk samples sintered at temperatures below 660 K show high similarity and uniformly exhibit a typical alternating lamellar feature, as displayed in Figure 6a–c. However, a distinct and significant microstructural transformation can be clearly identified when the sintering temperature rises to 660 K. Compared with the lamellar structure formed at lower sintering temperatures, the microstructure of the samples sintered at 660 K evolves into a much finer, more uniformly distributed refined lamellar architecture, as observed in Figure 6d.

**Figure 7:**
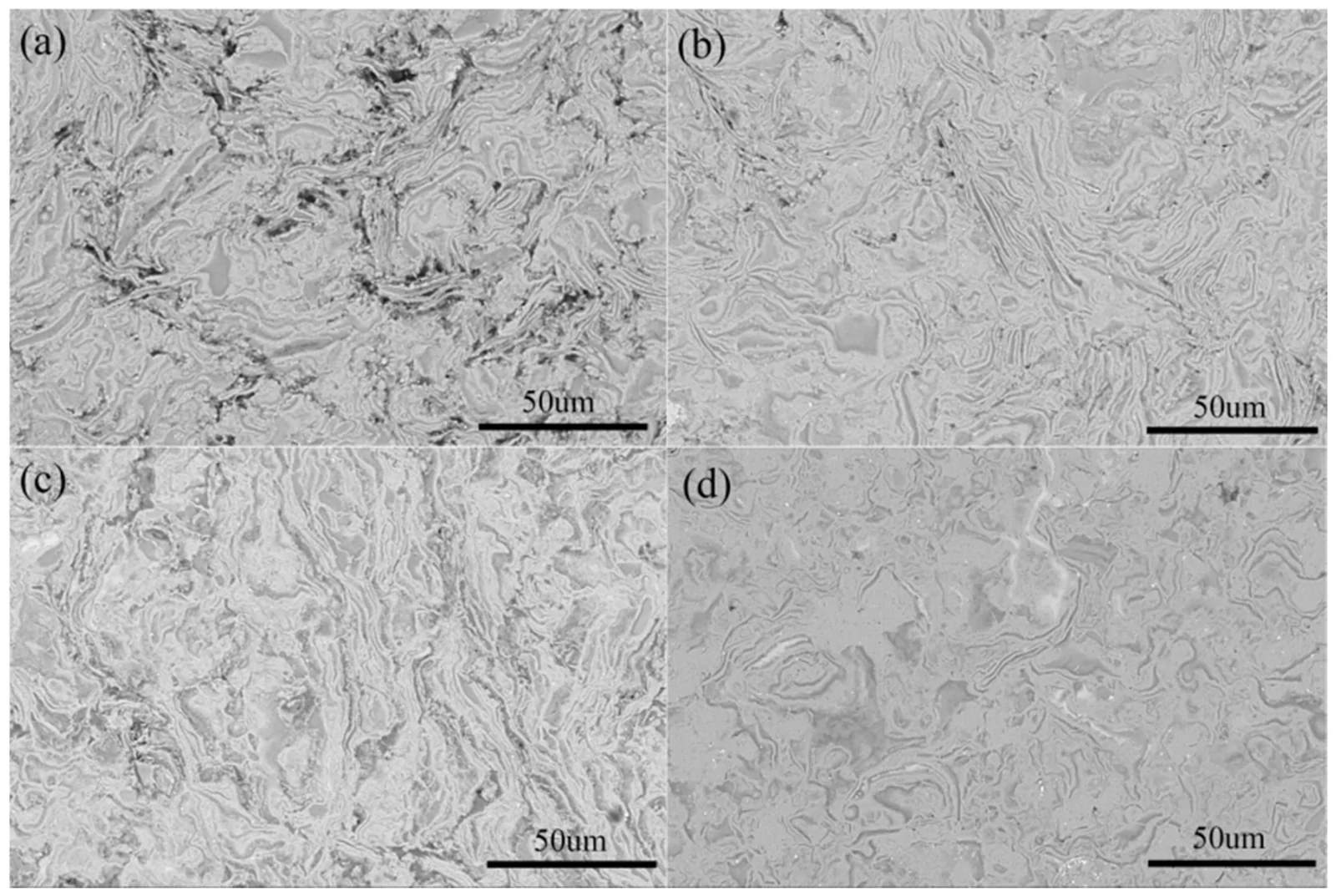
SEM morphology of the bulk samples sintered at different temperatures. (**a**) 450 K, (**b**) 510 K, (**c**) 580 K, (**d**) 660 K.

Furthermore, scanning electron microscopy (SEM) characterization was performed on the mirror-polished cross-sections of the sintered bulk composites to systematically observe the internal micro-defects and the interfacial bonding state between the layered structural units. The SEM micrographs of the bulk samples consolidated at different sintering temperatures are presented in Figure 7. The characteristic microstructural features observed in these SEM images show excellent consistency with the corresponding OM morphologies displayed in Figure 6.

It can be clearly identified that microscale pores, appearing as dark contrast regions in the backscattered electron mode, are distributed in all bulk samples sintered at different temperatures. Both the total number and the average size of these residual pores gradually decrease monotonically as the sintering temperature increases. Notably, these residual pores are preferentially located at the interfaces between the adjacent lamellar structural units. These observations directly confirm that the structural compactness of the bulk composites is significantly improved with the elevation of sintering temperature, which provides a direct microstructural explanation for the rising trend of bulk density and Vickers microhardness measured in previous tests. In addition, by comparing the micrographs of the 580 K and 660 K sintered samples in Figure 6c,d and Figure 7c,d, it can be further concluded that the remarkable microstructural refinement of the lamellar architecture acts as another critical strengthening mechanism, which further contributes to the higher microhardness of the 660 K sintered sample compared with the 580 K sintered counterpart.

**Figure 8:**
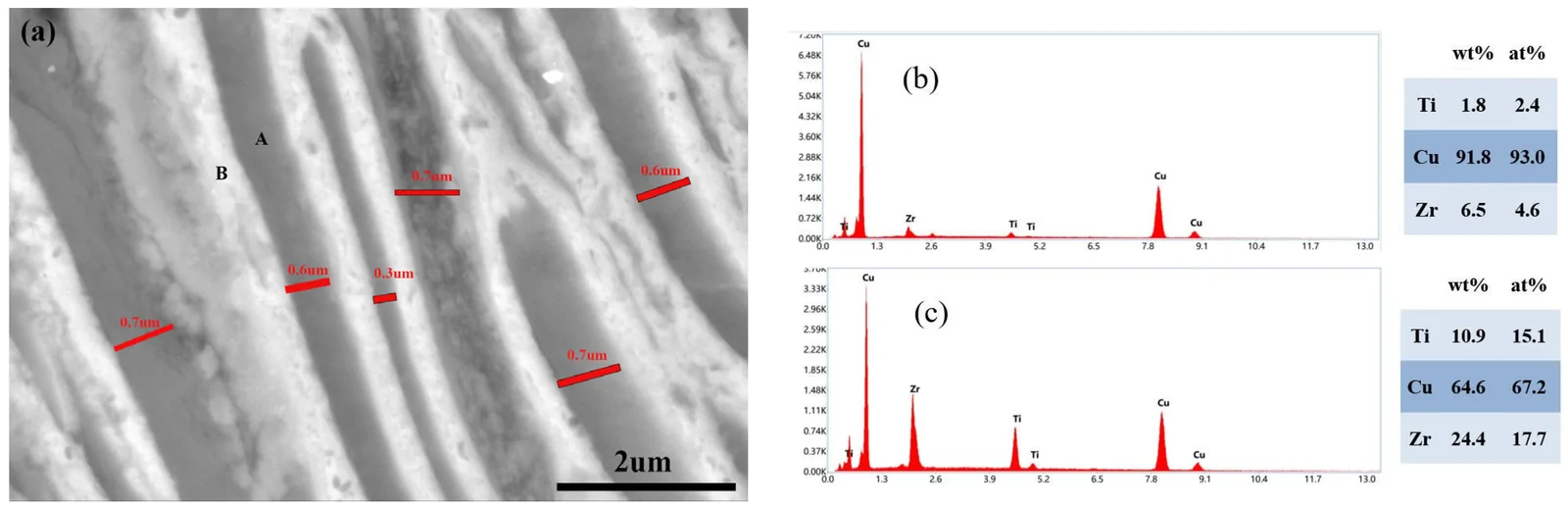
Typical lamellar structures for the bulk samples (**a**) and EDS results for A (**b**) and B (**c**).

Furthermore, the typical lamellar microstructure of the sintered composite is clearly presented in the backscattered electron SEM image in Figure 8. As observed, two distinct phases, i.e., the black phase and the white phase, are arranged alternately in the matrix. Combined with the EDS point scanning results, region A (the black phase in Figure 8b) is mainly composed of Cu, with only a small amount of dissolved Zr and Ti elements, while the composition of region B (the white phase in Figure 8c) is close to the nominal composition of the designed Cu-Zr-Ti alloy. This obvious composition deviation between the two phases originates from the interdiffusion of alloying elements driven by the localized high temperature effect during the spark plasma sintering (SPS) process. Based on the statistical measurement of more than 10 different SEM fields of view, the average thickness of the Cu coating layer in the sintered sample is estimated to be approximately 0.3 μm.

**Figure 9:**
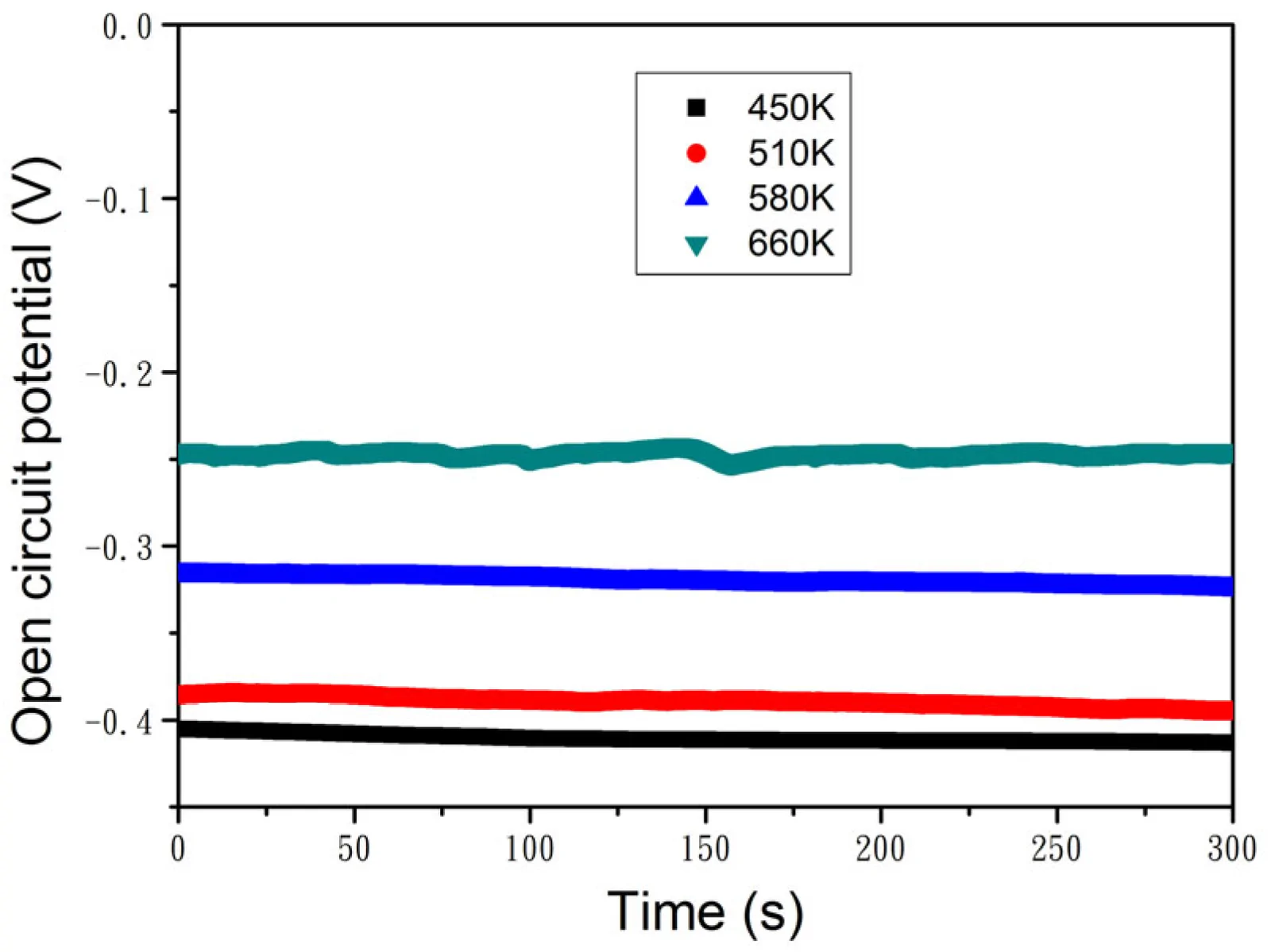
Dependence of open circuit potential on the duration for the samples sintered under different temperatures.

Figure 9 shows the evolution of the open circuit potential (E_OCP_) as a function of immersion time for the sintered bulk samples in 3.5 wt.% NaCl solution. As observed in the figure, the E_OCP_ of each sample gradually stabilizes to a steady-state value that is closely correlated with the sintering temperature. The corresponding steady-state E_OCP_ values after 30 min of immersion are summarized in Table 3, and the results show that the stabilized E_OCP_ increases progressively with the increase in sintering temperature.

**Figure 10:**
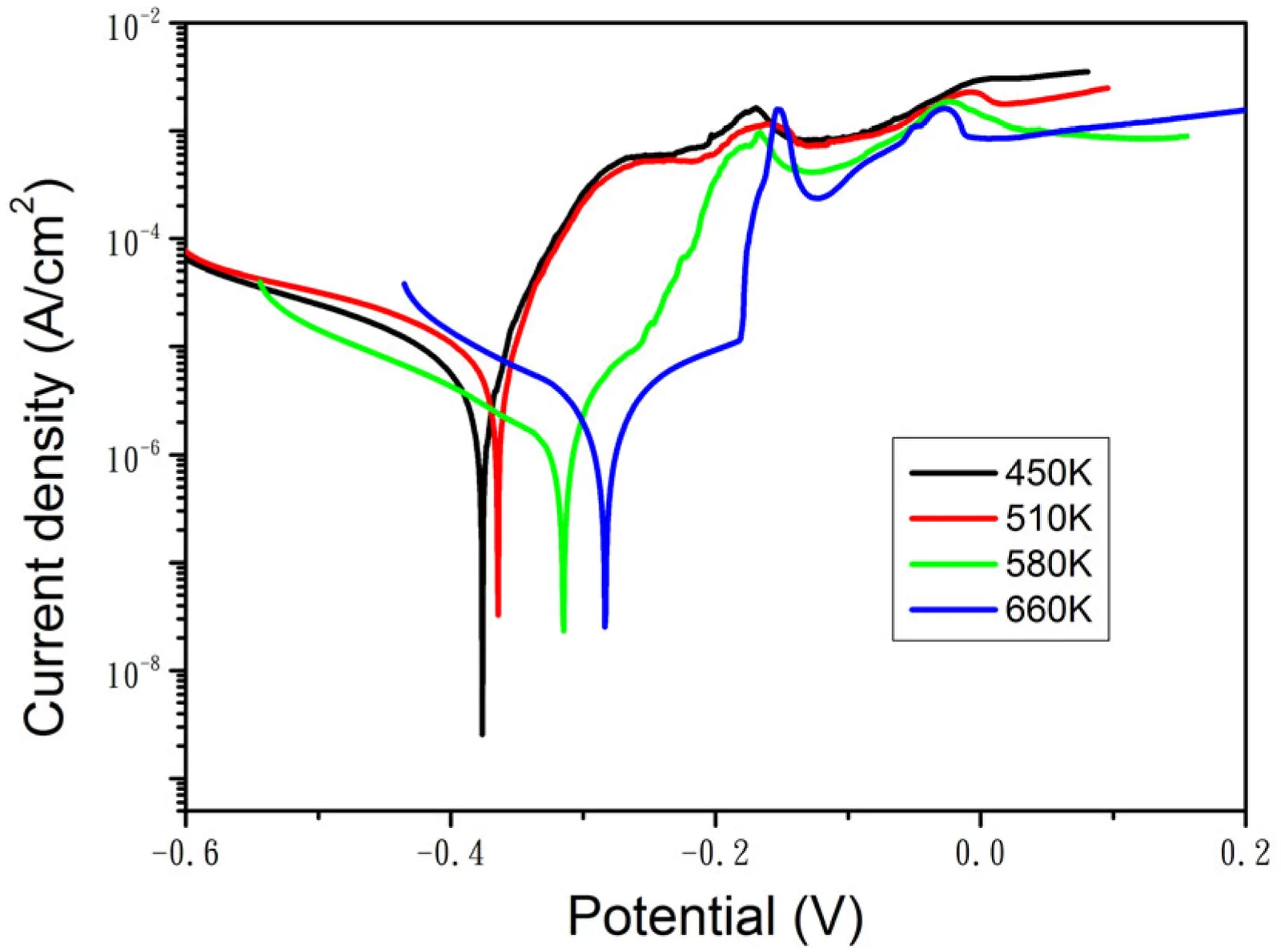
Polarization curves of the samples sintered under different temperatures in 3.5 wt.% NaCl solution.

In addition, the potentiodynamic polarization curves of the bulk sintered samples are presented in Figure 10. The curves of the samples sintered at 450 K and 510 K show highly consistent profiles, indicating that these two groups share nearly identical corrosion behavior. Similarly, the polarization curves of the 580 K and 660 K sintered samples also exhibit comparable features, suggesting their similar corrosion response. As observed in Figure 10, the three characteristic stages of anodic polarization take place—activation, passivation and pitting corrosion overlap with each other on the curves of the 450 K and 510 K samples—while these three stages are clearly distinguishable on the curves of the 580 K and 660 K groups. This difference directly demonstrates that the latter two samples can form a passive film much more easily, and the as-formed passive film also possesses better structural stability than that of the low-temperature sintered groups. Two distinct anodic peaks can be detected on all the polarization curves, which correspond to two metastable pitting events occurring within the tested potential range. Key electrochemical parameters, including corrosion potential (E_corr_), corrosion current density (i_corr_), passivation potential (E_p_), passivation current density (i_p_) and pitting potential (E_pit_), were extracted and calculated systematically, with all detailed values summarized in Table 3. The E_corr_ value increases progressively as the sintering temperature rises, which implies an improved thermodynamic corrosion stability of the sample. All measured i_corr_ values fall in the order of 10^−7^ A/cm^2^, confirming that the as-sintered composites possess excellent intrinsic corrosion resistance in 3.5 wt.% NaCl solution. The i_corr_ shows a continuous decreasing trend with the elevation of sintering temperature, further verifying the gradual enhancement of the overall corrosion resistance. Compared with the 580 K sintered sample, the 660 K-sintered sample exhibits a higher E_p_, which indicates that the passive film can be more stable. Meanwhile, the 660 K sintered sample has a smaller i_p_, and its passivation region is 2.6 times wider than that of the 580 K sintered counterpart, proving that the passive film formed on the high-temperature sintered sample has significantly better protective performance. Moreover, the 660 K sintered sample also delivers a higher E_pit_ than the 580 K group, which corresponds to a much lower pitting corrosion tendency. This trend of corrosion resistance improvement with increasing sintering temperature is well supported by previous studies on Zr-based bulk metallic glasses. Shin et al. [39] reported that the corrosion resistance of Zr_65_Al_10_Ni_10_Cu_15_ bulk metallic glass in 0.1 N HCl solution increased with the rising annealing temperature. A similar phenomenon was also observed by González et al. [40], who found that the corrosion resistance of Zr_60_Cu_20_Al_10_Fe_5_Ti_5_ bulk metallic glass in 1 M NaCl solution was progressively enhanced at higher annealing temperatures.

**Figure 11:**
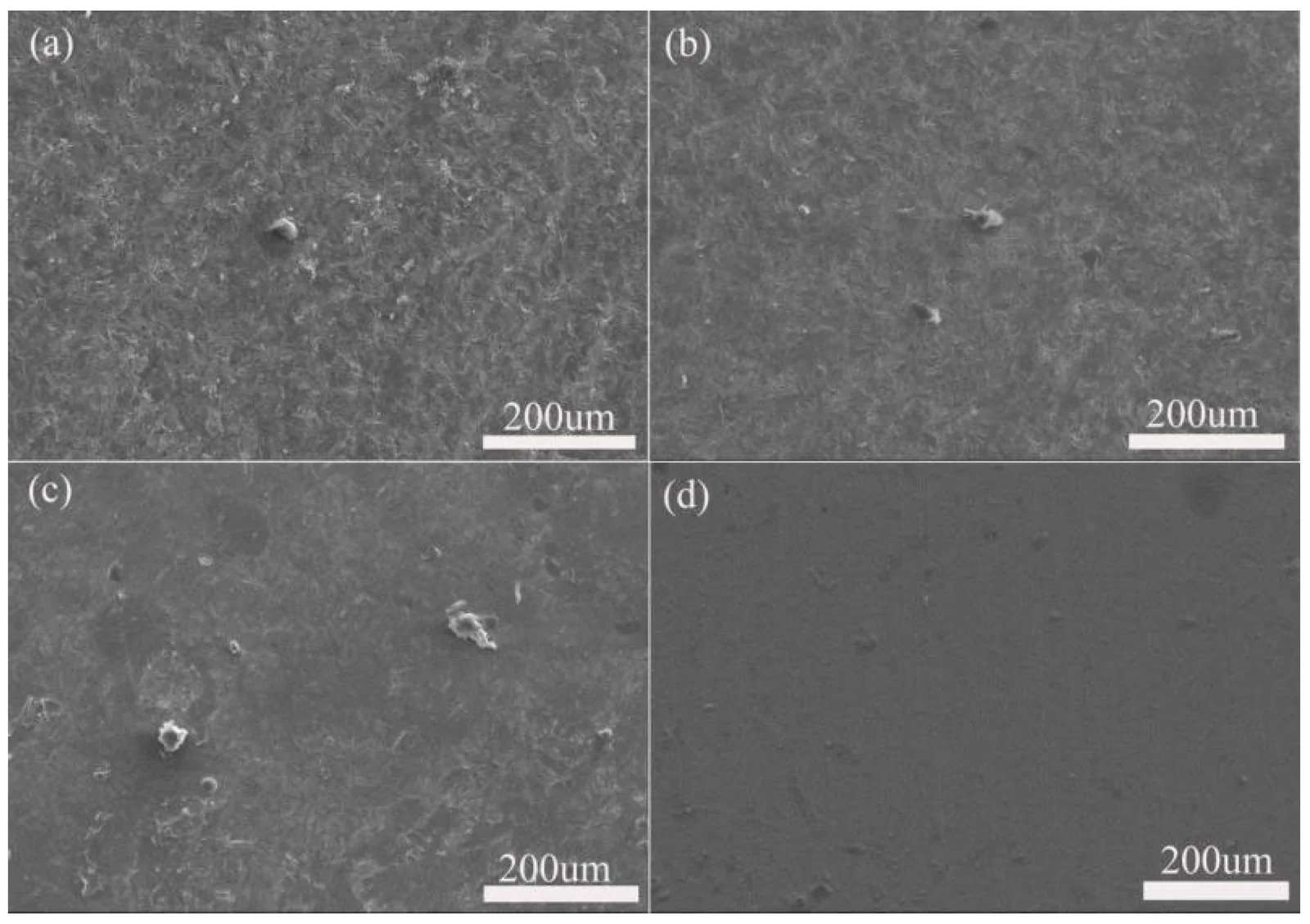
SEM images of the corroded surfaces of the sintered samples in 3.5 wt.% NaCl. (**a**) 450 K, (**b**) 510 K, (**c**) 580 K, (**d**) 660 K.

**Figure 12:**
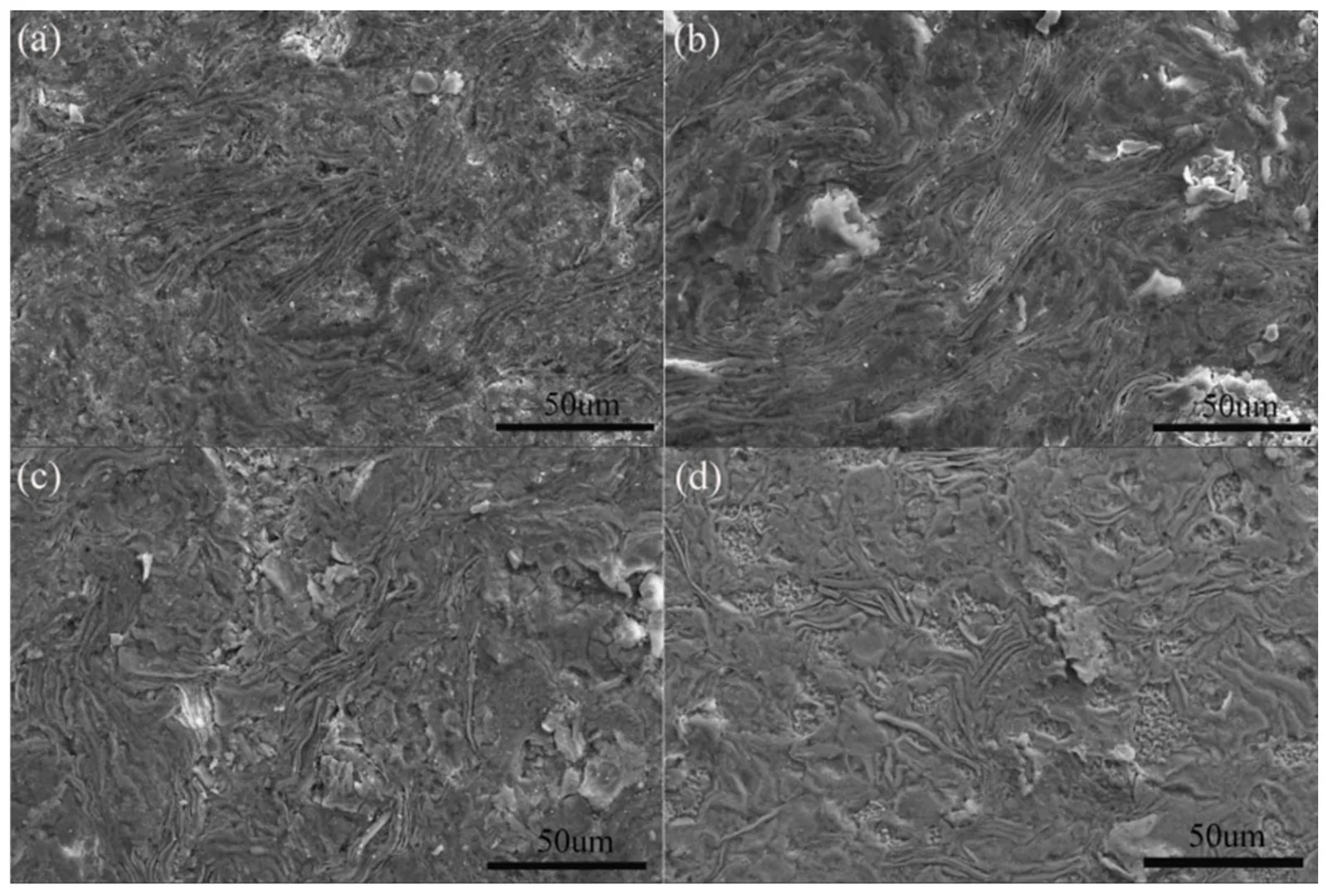
Local enlarged SEM images of the corroded surfaces of the sintered samples in 3.5 wt.% NaCl. (**a**) 450 K, (**b**) 510 K, (**c**) 580 K, (**d**) 660 K.

Furthermore, the corroded surface morphologies of the bulk samples were characterized by scanning electron microscopy (SEM). Figure 11 and Figure 12 display the typical SEM micrographs of the corroded surfaces. As observed in Figure 11, corrosion products and etching pits are clearly visible on all sample surfaces. Both the coverage of corrosion products and the number of etching pits gradually decrease as the sintering temperature rises, and the corroded surface of the 660 K sintered sample is significantly smoother than that of the other groups. The high-magnification SEM images in Figure 12 further confirm that both the quantity and average size of the etching pits show a downward trend with increasing sintering temperature. For the samples sintered at 450 K and 510 K, only local, sporadic passive films can be observed on the corroded surfaces. In contrast, continuous and intact passive films form on the surfaces of the 580 K and 660 K sintered samples, and the passive film on the 660 K sintered sample exhibits higher density and better continuity than that on the 580 K counterpart. These morphological results directly prove that both the corrosion resistance and pitting resistance of the samples are progressively enhanced with the elevation of sintering temperature, which is fully consistent with the potentiodynamic polarization test results. This improvement can be attributed to the fact that higher sintering temperature effectively increases the compactness and integrity of the as-formed passive film, and it is also closely related to the difference in crystallization degree of the sintered samples under different temperatures (see Table 2). This trend is well supported by existing studies on Zr-, Ni- and Ti-based metallic glasses. Cai et al. [41] and Shin et al. [39] reported that the chloride corrosion resistance of Zr-based bulk metallic glasses could be significantly improved when the sintering temperature was controlled within an appropriate range. González et al. [40] demonstrated that for Zr-based bulk metallic glasses tested in 1 M NaCl solution, the corrosion resistance increased with the rising annealing temperature, and the annealed samples exhibited better corrosion performance than the as-cast counterparts. Zhao et al. [42] found that the crystallized Ni_62_Nb_33_Zr_5_ bulk metallic glass possessed higher corrosion resistance than its fully amorphous state in 3.5 wt.% NaCl solution. Fornell et al. [43] confirmed that the devitrified Ti_40_Zr_10_Cu_38_Pd_12_ metallic glass showed superior corrosion resistance in Hank’s solution compared with the fully amorphous sample. Perumal et al. [44] also verified that appropriate low-temperature annealing treatment could effectively enhance the corrosion resistance of Zr_65.7_Cu_15.6_Ni_11.7_Al_3.7_Ti_3.3_ metallic glass in Ringer’s solution.

**Figure 13:**
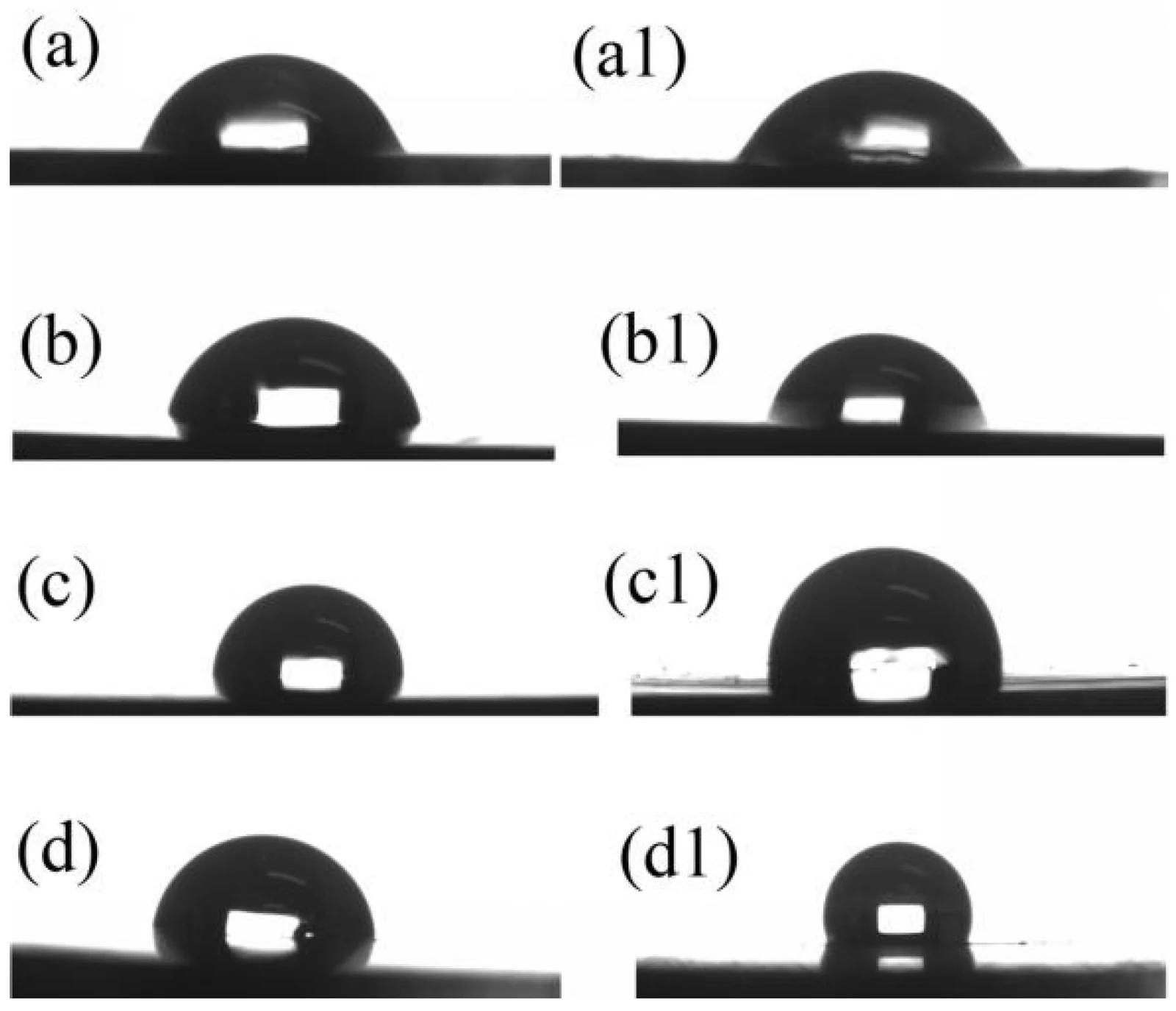
Wetting behavior of the mirror-polished samples under different sintering temperatures. (**a**) 450 K, (**b**) 510 K, (**c**) 580 K, (**d**) 660 K. (**a1**–**d1**) are the corresponding corroded surface.

The surface wettability of the polished and corroded samples was assessed aiming to the marine service of the studied Cu alloys. The wetting behavior of the bulk sintered composites was characterized via static water contact angle measurements on the sample surfaces using deionized water. Figure 13 presents the wetting performance of both as-sintered and corroded samples, and Table 4 summarizes the corresponding contact angle values for the two types of surfaces. As the sintering temperature rises, the contact angle increases from 77.4° to 104.2° for the polished surfaces and from 75.7° to 111.9° for the corroded surfaces. This indicates that the wetting state of both polished and corroded surfaces transforms from hydrophilicity to hydrophobicity with increasing sintering temperature. Notably, for the samples sintered at 450 K and 510 K, the contact angles of the polished surfaces are larger than those of their corresponding corroded counterparts. However, for the 580 K and 660 K sintered samples, the contact angles increase after the corrosion test. The contact angle values of the studied alloy are comparable to those of reported Cu-based amorphous alloys, such as the smooth surface of Cu_48_Zr_42_Ti_4_Al_6_ (106.6°), the smooth surface of Cu_47_Zr_42_Al_7_Ti_4_ (83.0°), and the wrinkled surface of Cu_50_Zr_50_ (72.3~112.8°) [45]. It is widely recognized that the wettability of amorphous alloys is governed by multiple factors, including alloy composition, surface topography, crystallization degree and surface chemical state [41,44,45,46,47,48,49,50]. Although the nominal composition of the initial composite alloy powders is identical, the local composition of the as-sintered bulk samples still shows distinct differences from each other. Higher sintering temperatures can effectively promote the interdiffusion of alloying elements, enabling Zr and Ti atoms in the Cu-Zr-Ti alloy to diffuse more sufficiently into the Cu coating, which finally leads to the local composition heterogeneity. Furthermore, the surface chemical composition of the corroded samples was semi-quantitatively analyzed by EDS, and the detailed results are listed in Table 5. As the sintering temperature increases, the atomic percentages of Cu and Cl decrease, while those of Zr, Ti and O show a gradual upward trend. This trend of wettability variation is consistent with several reported studies on metallic glasses. Perumal et al. [44] confirmed that appropriate low-temperature annealing could increase the contact angle of Zr_65.7_Cu_15.6_Ni_11.7_Al_3.7_Ti_3.3_ metallic glass. However, Cai et al. [41] found an opposite trend that the contact angle decreased with rising sintering temperature for spark-plasma-sintered Zr_48_Cu_47.5_Al_4_Co_0.5_ amorphous alloy composites. This discrepancy further indicates that the wettability of amorphous alloys is a comprehensive result of multiple influencing factors. In addition, Figure 6 and Figure 7 demonstrate that the surface topography of the as-sintered samples varies significantly, including the morphology of the layered structure, as well as the size, dimension and distribution of internal pores. Xiao et al. [46] reported that the wettability of Zr_55_Cu_30_Ni_5_Al_10_ bulk metallic glass surface was deteriorated after electrochemical corrosion, and the hydrophobicity could be further enhanced by constructing micro-nano hierarchical structures via stearic acid modification. Hasan et al. [50] observed that Pd_43_Cu_27_Ni_10_P_20_ metallic glass remained in a hydrophilic state regardless of its surface topography, which was attributed to the spontaneous surface oxidation in an ambient environment.

## 4. Discussion

As summarized in Figure 13 and Table 4, the static water contact angles of the 450 K and 510 K sintered samples show a distinct decreasing trend after corrosion, while those of the 580 K and 660 K sintered counterparts exhibit an opposite increasing tendency. This divergent variation behavior can be attributed to the combined effect of two key factors: the evolution of surface micro-topography and the modification of surface chemistry, which has been widely reported in previous studies [41,44,45,46,47,48,49,50].

The 450 K and 510 K sintered samples are intrinsically hydrophilic in their original polished state. According to the classic Wenzel wetting model [51], the apparent contact angle θ_w_ follows the relationship cos θ_w_ = rcos θ, where r is the surface roughness factor defined as the ratio of actual solid–liquid contact area to the projected geometric area, and θ is the intrinsic contact angle of the smooth, flat sample surface. After the corrosion test, the surface roughness of these two low-temperature sintered samples increases significantly, originating from the formation of loose corrosion products and the generation of micro-scale corrosion pits, as clearly observed in the post-corrosion SEM morphologies in Figure 11 and Figure 12. For intrinsically hydrophilic materials where the intrinsic contact angle θ < 90°, an increase in roughness will theoretically amplify the hydrophilicity and decrease the apparent contact angle recorded in the test.

The 660 K sintered sample maintains an intrinsic hydrophobic nature, as verified by the original contact angle data in Figure 13 and Table 4. After corrosion, the increased surface roughness further amplifies its hydrophobicity, leading to the elevated apparent contact angle recorded in the test. For the 580 K sintered sample, which belongs to the intrinsically hydrophilic system, the roughness-enhancement mechanism alone cannot explain the experimentally observed increase in contact angle, since the measured post-corrosion surface roughness would theoretically reduce its apparent contact angle according to the Wenzel model. This contradiction strongly implies that the surface chemistry plays a dominant regulatory role in its final wetting behavior.

**Figure 14:**
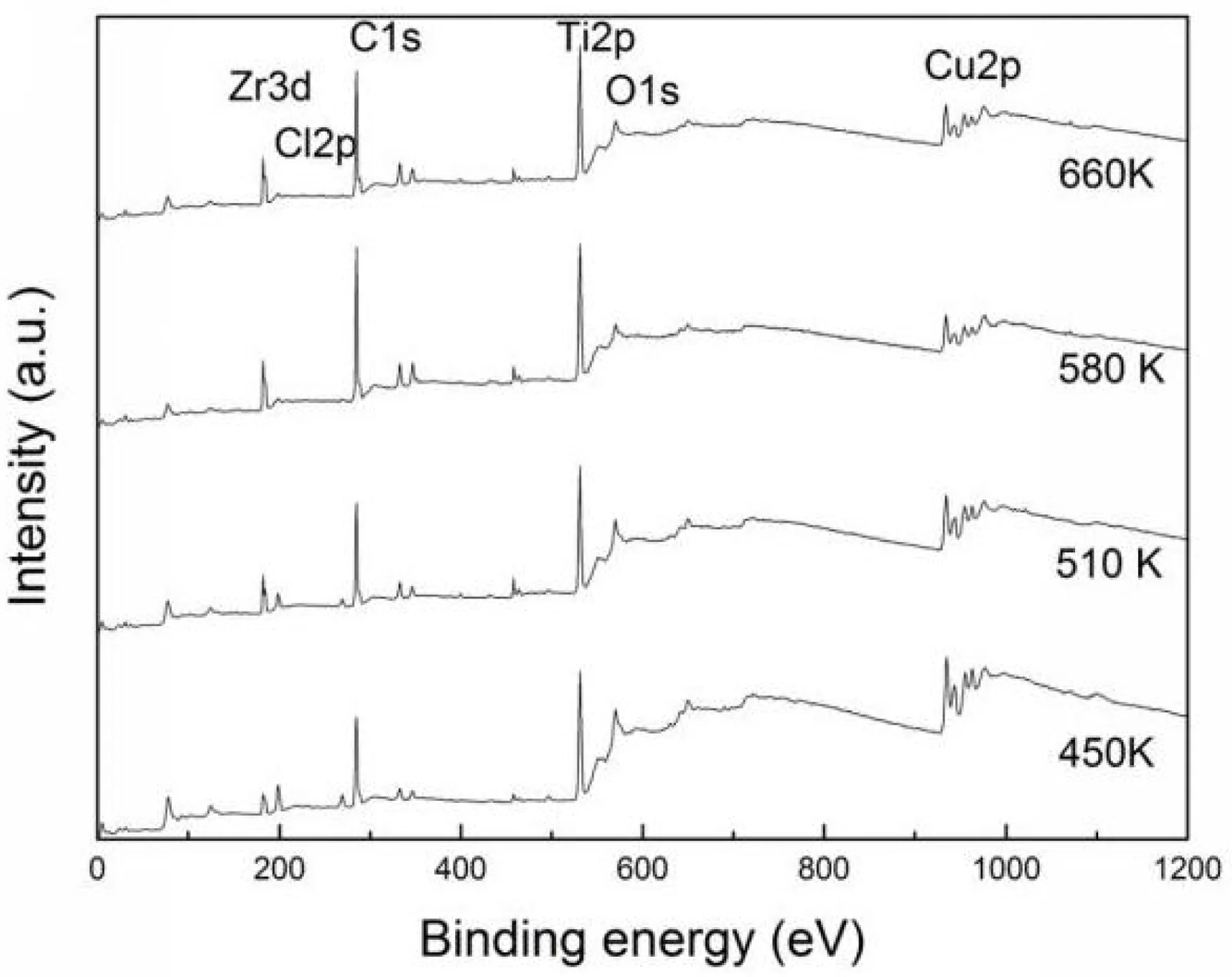
XPS full spectra of the corroded surfaces at different sintering temperatures.

To further clarify the surface chemical state of the corroded samples, X-ray photoelectron spectroscopy (XPS) characterization was performed on all post-corrosion surfaces. The full-scan XPS spectra of the representative samples for surface chemical analysis are presented in Figure 14, and the corresponding atomic percentages of different elements detected on the outermost passive layer are summarized in Table 6. The full-scan XPS spectra of all four samples show highly similar peak distributions, indicating that the overall elemental composition of the surface passive film formed after corrosion is nearly identical across all sintering conditions.

**Figure 15:**
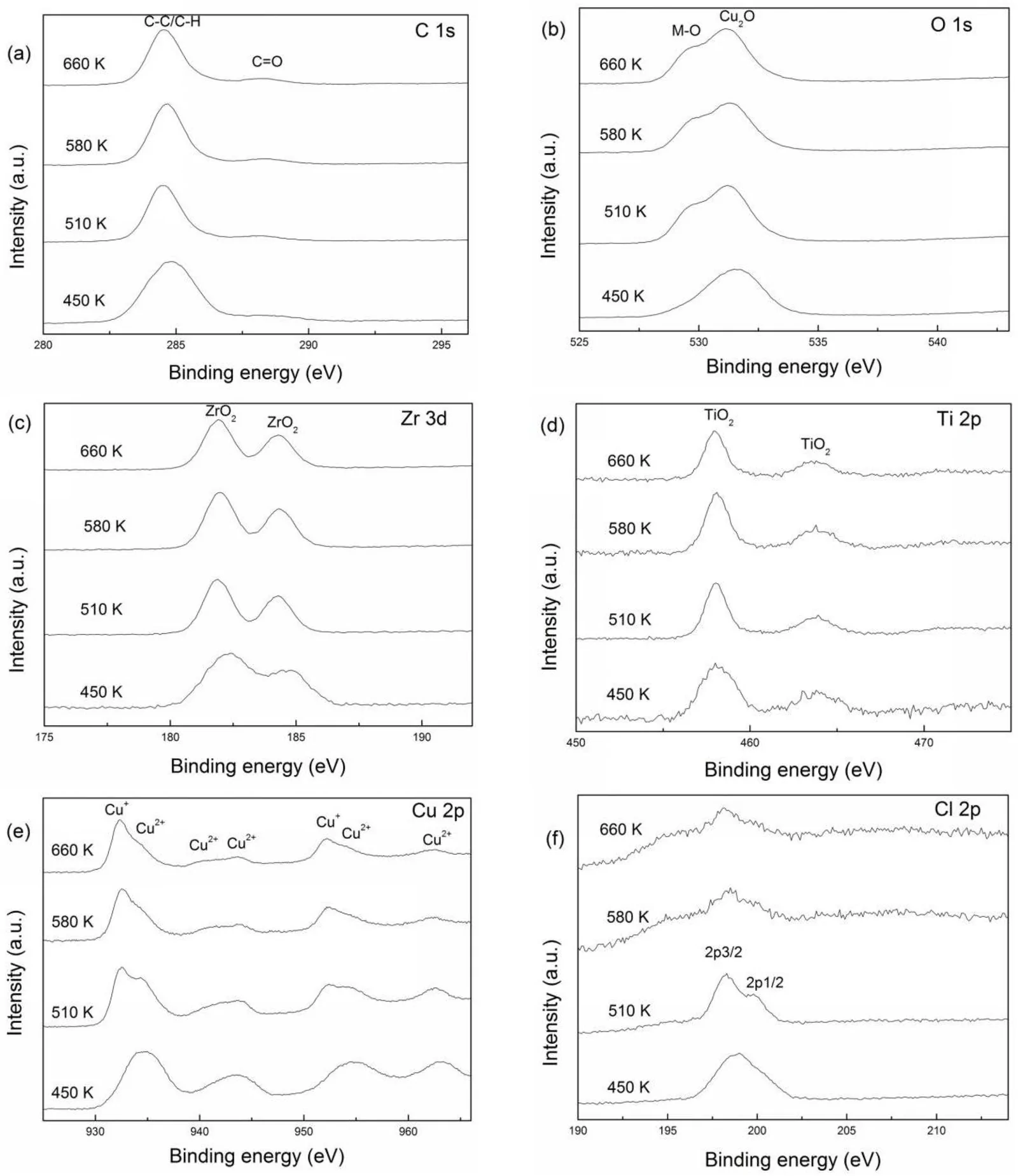
XPS high-resolution spectra of the detected alloy elements of the corroded surfaces at different sintering temperatures. (**a**) C 1s; (**b**) O 1s; (**c**) Zr 3d; (**d**) Ti 2p; (**e**) Cu 2p; (**f**) Cl 2p.

Figure 15 displays the high-resolution narrow-scan XPS spectra of all major detected elements collected from the corroded sample surfaces. The C 1s spectrum (Figure 15a) can be deconvoluted into two distinct fitted peaks located at 284.8 eV and 288.5 eV, which are assigned to the C-C/C-H adventitious carbon species and C=O carbon-oxygen functional groups, respectively. The O 1s spectrum (Figure 15b) is also well fitted by two separated peaks centered at 529.8 eV and 531.5 eV, which correspond to the metal–oxygen (M-O) lattice oxide bonds and cuprous oxide (Cu_2_O) species, respectively. The Zr 3d high-resolution spectrum in Figure 15c exhibits a characteristic spin-orbit doublet, where the 3d_5/2_ component at 182.5 eV and the 3d_3/2_ component at 184.5 eV can be unambiguously attributed to the fully oxidized Zr^4+^ chemical state. As shown in Figure 15d, the Ti 2p spectrum presents a typical spin-orbit doublet feature, with the 2p_3/2_ peak located at 458.5 eV and the 2p_1/2_ peak at 464.0 eV, both of which originate from the Ti^4+^ oxidized state. The high-resolution Cu 2p spectrum in Figure 15e unambiguously confirms the coexistence of Cu^2+^ and Cu^+^ chemical states on the corroded composite surface. The weak but distinguishable satellite shake-up features characteristic of divalent copper, combined with the corresponding Cu_2_O and CuO oxygen species signals identified in the O 1s spectrum, eliminate the common misinterpretation caused by binding energy overlap between metallic Cu and Cu^+^, providing solid cross-validated evidence for the multi-valence distribution of surface copper. The Cl 2p spectrum (Figure 15f) shows a single set of spin-orbit doublet peaks, where the 2p_3/2_ component at 198.6 eV and the 2p_1/2_ component at 199.6 eV are assigned to the Cl^−^ ionic state.

It can be observed from Table 6 that carbon (46.8~54.5 at.%) and oxygen (32.0~33.0 at.%) are the dominant elements detected on all corroded surfaces, which can be primarily attributed to ubiquitous adventitious surface contamination and native oxidation of the composite matrix. In addition, characteristic signals of Cu, Zr and Ti originating from the bulk alloy, as well as Cl element introduced by the 3.5 wt% NaCl corrosion medium, are unambiguously identified across all tested samples. As the sintering temperature increases, the relative atomic fractions of Cu and Cl show a monotonic decreasing trend, while the contents of Zr, Ti, O and C gradually rise, which is in excellent agreement with the corresponding EDS results summarized in Table 5.

**Figure 16:**
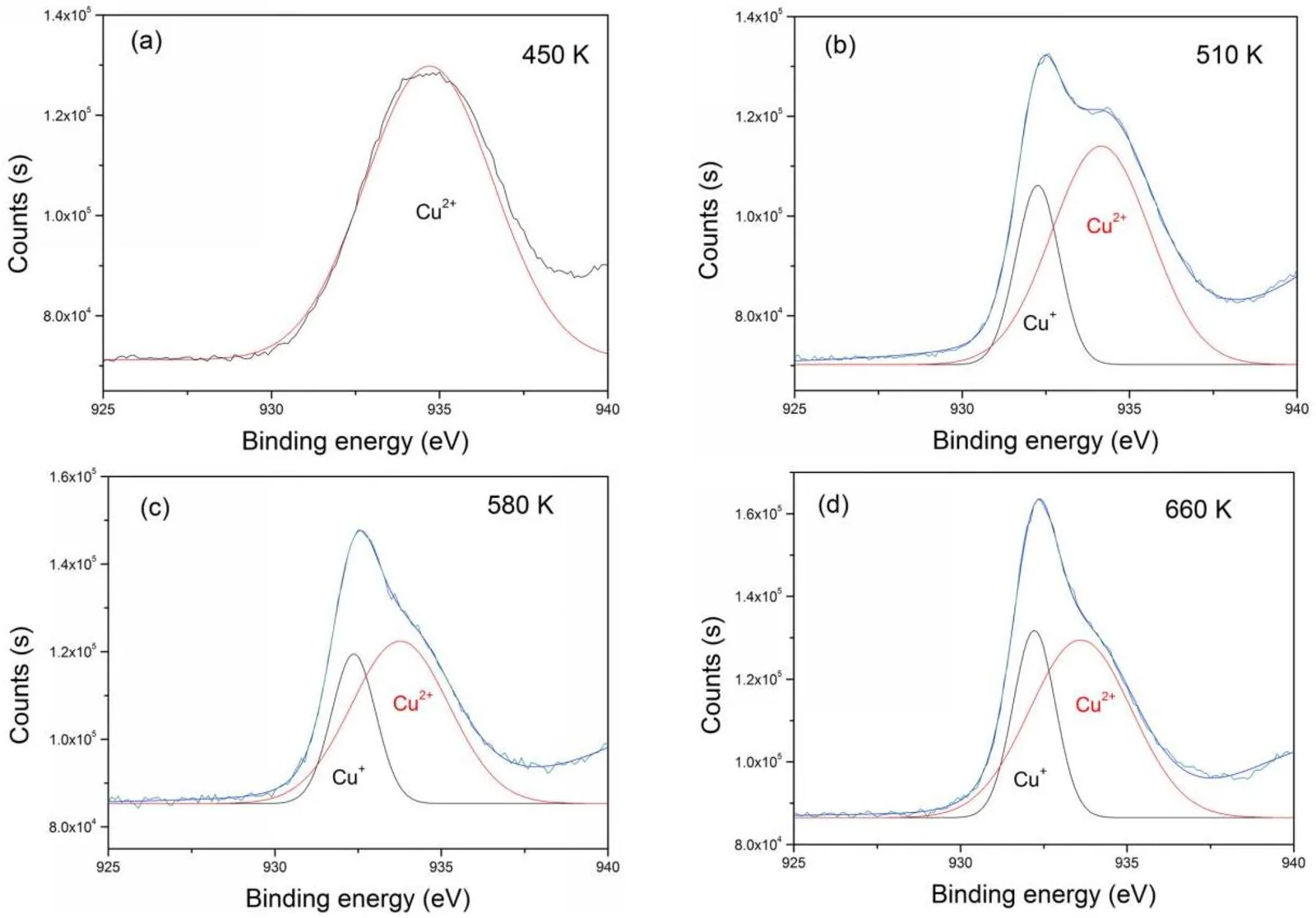
Deconvoluted core-level spectra of Cu 2p of first main peak for the corroded surfaces at different sintering temperatures. (**a**) 450 K; (**b**) 510 K; (**c**) 580 K; (**d**) 660 K.

**Figure 17:**
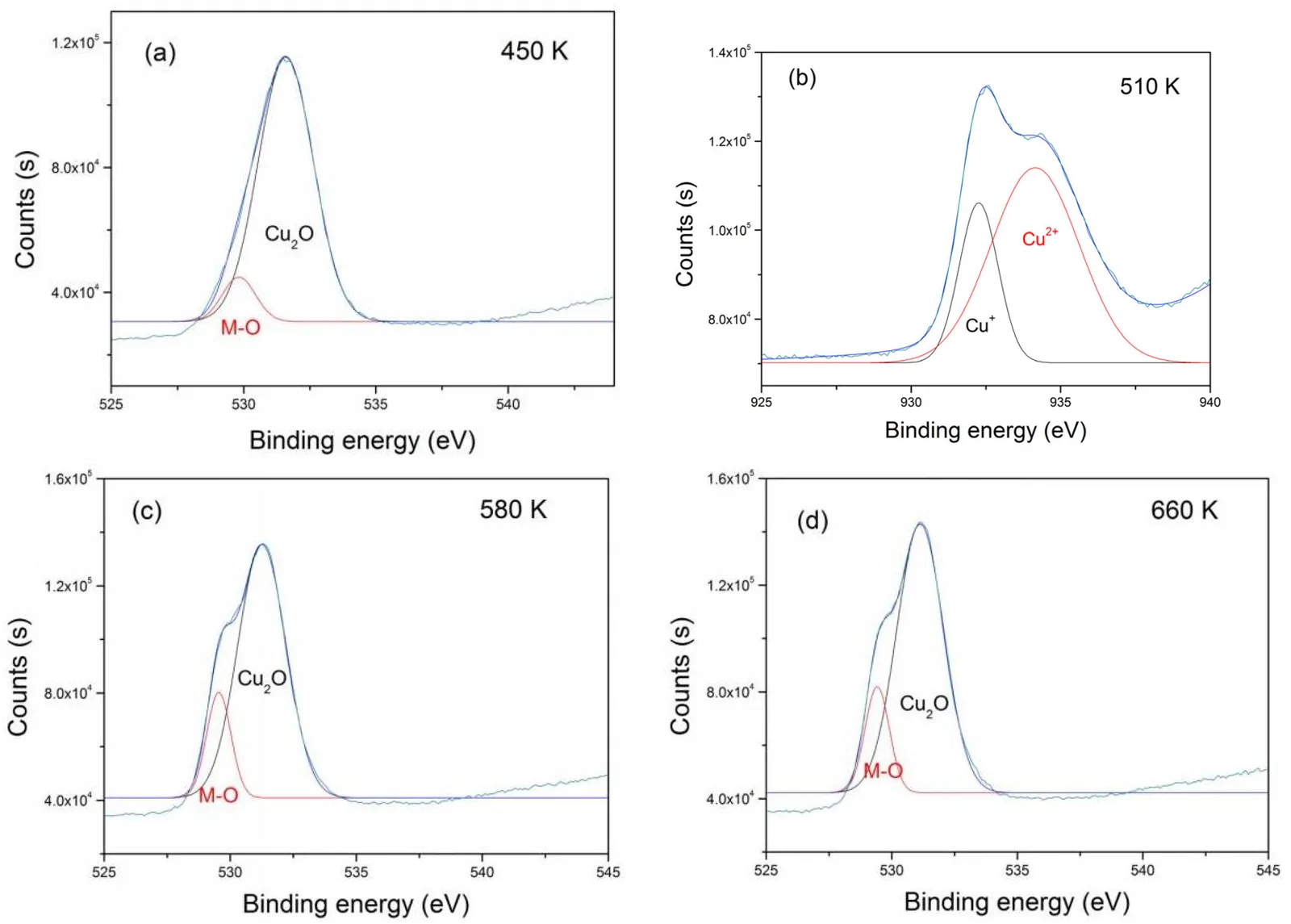
Deconvoluted core-level spectra of O 1s for the corroded surfaces at different sintering temperatures. (**a**) 450 K; (**b**) 510 K; (**c**) 580 K; (**d**) 660 K.

**Figure 18:**
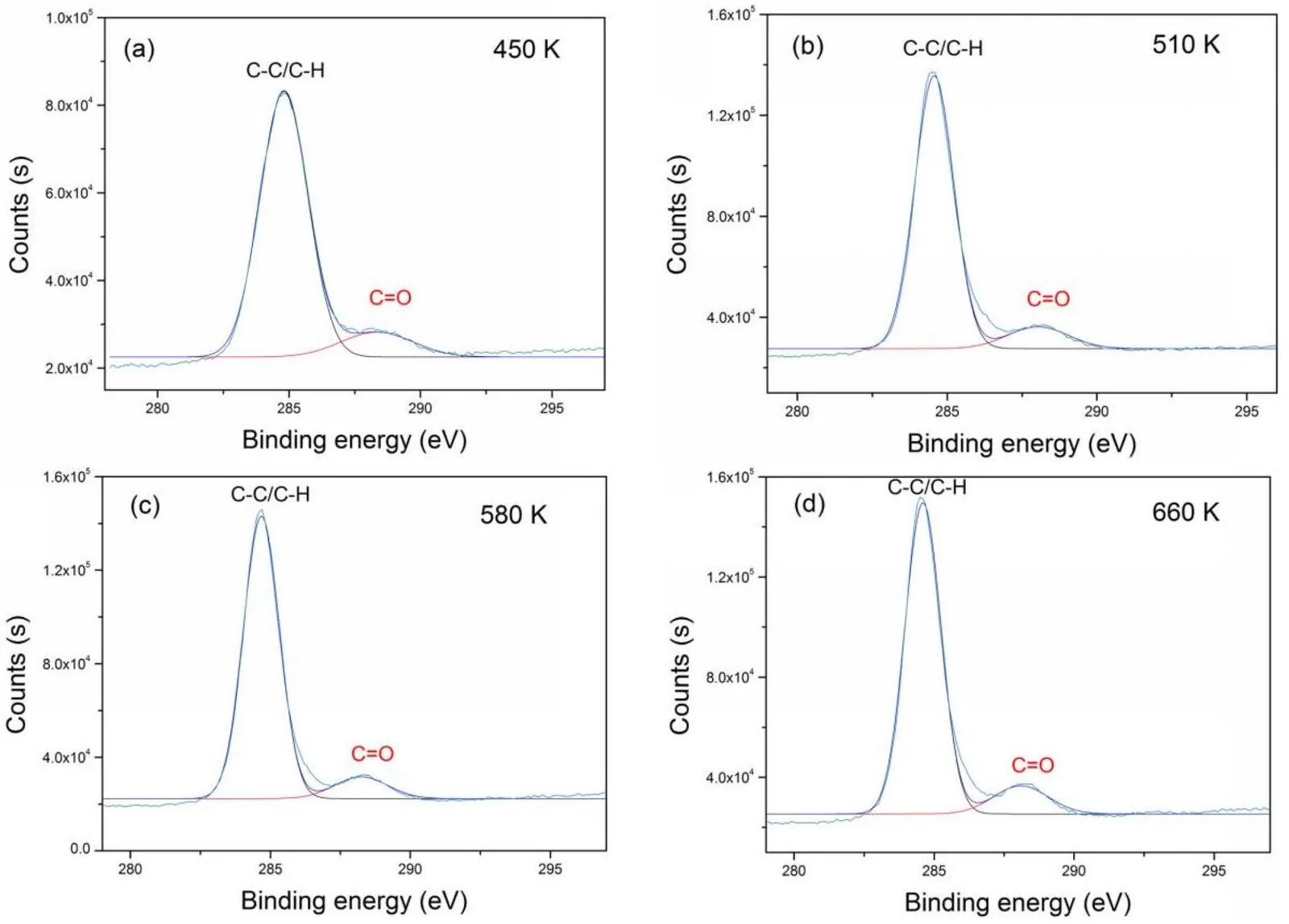
Deconvoluted core-level spectra of C 1s for the corroded surfaces sintered at different temperatures. (**a**) 450 K; (**b**) 510 K; (**c**) 580 K; (**d**) 660 K.

It can be further inferred from the combined analysis of Figure 14 and Figure 15 and Table 6 that the relative content of ZrO_2_ and TiO_2_ in the surface passive layer increases with elevated sintering temperature, whereas the fraction of copper chloride corrosion products decreases correspondingly. To quantitatively resolve the content variation of different copper oxide species, high-resolution Cu 2p and O 1s core-level spectra were systematically deconvoluted. As illustrated in Figure 16 and Figure 17, the relative proportion of CuO decreases monotonically, while that of Cu_2_O increases steadily as the sintering temperature rises. The deconvoluted C 1s core-level spectra are presented in Figure 18, where the peak intensity assigned to non-polar C-C/C-H groups gradually increases, while the intensity of polar C=O functional groups shows a consistent decreasing trend with increasing sintering temperature.

It has been widely reported in previous studies that ZrO_2_, TiO_2_ and copper chloride are intrinsically hydrophilic materials that can effectively enhance the surface hydrophilicity [52]. Consistent with the above characterization results, the rising fraction of ZrO_2_ and TiO_2_ at higher sintering temperatures would theoretically contribute to improved surface hydrophilicity. However, the simultaneous decrease of copper chloride content would exert an opposite effect and reduce the surface hydrophilicity. Further investigations confirm that non-polar C-C(H) covalent bonds are associated with hydrophobic surface behavior, while polar C=O groups tend to increase surface hydrophilicity [47,53]. The observed trend of increasing C-C/C-H fraction and decreasing C=O fraction with higher sintering temperature would therefore promote the hydrophobicity of the corroded surface. In addition, Cu_2_O is verified to exhibit inherent hydrophobic properties, while CuO shows highly hydrophilic characteristics [49,54]. The continuous increase of the Cu_2_O fraction and decrease of the CuO fraction at elevated sintering temperatures would further enhance the surface hydrophobicity.

Consequently, the experimentally observed monotonic increase of water contact angles on corroded surfaces with rising sintering temperature (see Table 4) can be rationally interpreted by the synergistic effect of the increased fractions of hydrophobic C-C/C-H groups and Cu_2_O species. Moreover, the reduction of water contact angles after corrosion for samples sintered at 450 K and 510 K, compared with their corresponding polished original surfaces, can be attributed to two main factors: the formation and increased fraction of hydrophilic ZrO_2_, TiO_2_, copper chloride and CuO species during the 3.5 wt% NaCl corrosion process, and the roughness amplification effect described by the Wenzel model, since the original polished surfaces of these two samples are intrinsically hydrophilic. For the 660 K sintered sample, the increase of water contact angle after corrosion is dominated by the formation and enrichment of hydrophobic Cu_2_O and C-C/C-H species, which counteracts the Wenzel roughness effect, as the original polished surface of this sample is intrinsically hydrophobic. For the 580 K sintered sample, the slight increase in water contact angle after corrosion is mainly governed by the formation and increased fraction of hydrophobic Cu_2_O and C-C/C-H groups.

**Figure 19:**
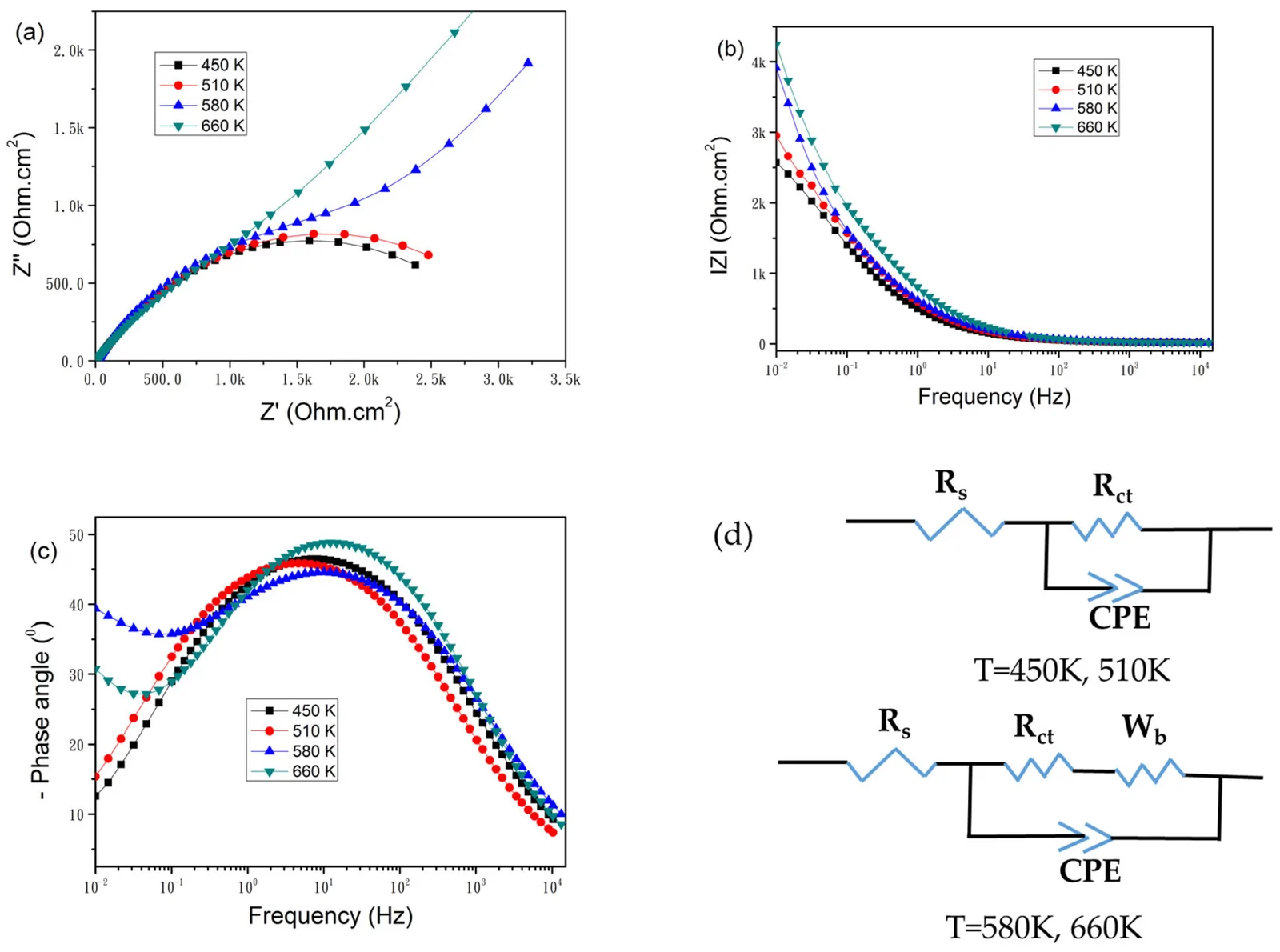
(**a**) Nyquist plots, (**b**) Bode magnitude plots, (**c**) Bode phase angle plots, (**d**) equivalent circuit model, respectively.

It is well known that the corrosion property and corrosion mechanism of the working electrode can be obtained through analyzing the electrochemical impedance. The electrochemical impedance spectroscopy (EIS) measurements were conducted on the bulk samples under different sintering temperatures in 3.5 wt.% NaCl solution at open circuit potentials. Figure 19 shows the Nyquist plots (Figure 19a), Bode magnitude plots (Figure 19b), Bode phase angle plots (Figure 19c), and the equivalent circuits (Figure 19d) for the samples under different sintering temperatures, respectively. The fitted results are in good agreement with the corresponding experimental data, indicating the rationality of the fitting. As shown in Figure 19a, the Nyquist diagrams are the capacitive reactance semicircles with no inductive arcs for the two samples sintered at 450 K and 510 K, indicating that the corrosion process is controlled by the charge transfer process [55]. The larger diameter suggests the higher resistance of the working electrode [56]. The diameter is larger for the 510 K sintered sample than for the 450 K sintered one, indicating that the corrosion resistance is better for the former than for the latter. On the other hand, the Nyquist diagrams obtained from the samples sintered at 580 K and 660 K present the capacitive reactance semicircles in the high-frequency region and the low-frequency diffusion tails associated with Warburg impedance in the low-frequency region. It means that the corrosion process is mainly controlled by the diffusion process. Next, we turn our attention to the Bode plots to further investigate the corrosion characteristics. As seen from the Bode magnitude plots of Figure 19b, the impedance modulus at high frequency is almost the same for all samples, indicating their same electrolyte resistance. However, the impedance modulus at low and intermediate frequency increases with the increase of the sintering temperature. It implies that the corrosion resistance increases with the increase of the sintering temperature. Moreover, equivalent circuit is used to simulate the EIS plots and presents in Figure 19d. The circuits comprise several key components: a solution resistance (R_s_), which accounts for the resistance of the electrolytic solution; a charge-transfer resistance (R_ct_), representing the resistance encountered during charge transfer at the electrode interface; a Warburg component (W_b_), reflecting the diffusion process’s contribution to the overall impedance; and a constant phase element (CPE), describing the non-ideal capacitive behavior due to electrode surface inhomogeneity. The fitted electrochemical parameters are listed in Table 7. The goodness of fit for all samples is controlled within the range of 10^−4^ to 10^−3^, and the fitting error of all elements is less than 5%. The total interfacial resistance (R_t_), which includes R_s_, R_ct_ and W_b_, increases with the increase in sintering temperature. It indicates that both the corrosion resistance and the quality of the passive film improve as the sintering temperature increases [55,57]. As shown in Figure 15c,d, Zr^0^ and Ti^0^ metals were not detectable. This indicates that the passive film was thick enough to prevent the detection of Ti and Zr metal contributions in all the studied corroded samples. In addition, it can be observed from Figure 15 and Table 6 that the ZrO_2_ and TiO_2_, which are known to have excellent corrosion resistance [52], increase as the sintering temperature rises. It can be seen from Figure 11 and Figure 12 that the integrity and compactness of the passive films increase as the sintering temperature increases. The aforementioned results explain why there is a transition from the charge-transfer process to the diffusion process and why the corrosion resistance increases as the sintering temperature rises.

On the other hand, numerous studies have demonstrated that the corrosion behavior of amorphous alloys is closely correlated with their surface wettability. It is widely accepted that hydrophobic surfaces play a positive role in improving the corrosion resistance of amorphous alloys [49,58,59,60,61,62]. For instance, Halder et al. [49] reported that the thicker atmospheric plasma-sprayed Fe_57_Cr_9_Mo_5_B_16_P_7_C_6_ coating was more susceptible to corrosion in 3.5 wt.% NaCl solution, which was attributed to its optimized porosity, pore size, hydrophobicity and crystallinity. Liang et al. [60] proposed that the superior corrosion resistance of arc-sprayed Fe_49.7_Cr_18_Mn_1.9_Mo_7.4_W_1.6_B_15.2_C_3.8_Si_2.4_ amorphous coatings in 3.5 wt.% NaCl solution was strongly correlated with their surface hydrophobicity. Xiao et al. [61] found that the corrosion resistance of Fe_48_Cr_15_Mo_14_C_15_B_6_Y_2_ amorphous alloy was significantly enhanced after constructing superhydrophobic surfaces via electrochemical corrosion and chemical modification. Wang et al. [62] pointed out that the better corrosion resistance of Zr_44_Ti_11_Cu_10_Ni_10_Be_25_ alloy in simulated body fluid compared with Zr_41_Ti_14_Cu_12_Ni_10_Be_23_ alloy originated from the higher surface hydrophobicity of the former. As illustrated in Figure 13 and Table 4, the water contact angles of the as-sintered samples show a monotonic increasing trend with the elevation of sintering temperature. This gradual enhancement of surface hydrophobicity acts as one of the critical contributing factors for the improved corrosion resistance observed in samples prepared at higher sintering temperatures. In addition, the elevated bulk density achieved at higher sintering temperatures (see Table 2) directly reduces the total exposed metallic surface area in contact with the corrosive electrolyte, which constitutes one of the fundamental mechanisms underlying the improved corrosion resistance of the sintered samples.

## 5. Conclusions

After 22 h of mechanical ball milling, the initial irregular block-like as-crushed Cu_70_Zr_15_Ti_15_ powders are transformed into flake-shaped amorphous composite powders. X-ray diffraction characterization confirms that the crystalline phases transforms from a single Cu_51_Zr_14_ to a Cu_51_Zr_14_+ amorphous dual-phase mixture. Subsequently, by carefully tuning the process parameters of electroless copper plating, the core-shell structured flake-shaped Cu_70_Zr_15_Ti_15_@Cu amorphous composite powders are successfully fabricated.

The dominant crystalline phase in the bulk sintered Cu_70_Zr_15_Ti_15_@Cu composites is metallic Cu. The average crystallite size (d) of the Cu phase shows an exponential dependence on the sintering temperature (*T*) within the tested temperature range of 450–660 K, and the corresponding fitting equation is: d = 22.036 − 20.233exp(−*T*/242.886). Both the bulk density (ρ) and Vickers microhardness (Hv) of the bulk composites increase monotonically with rising sintering temperature. The linear fitting relationships between these two properties and sintering temperature are expressed as ρ = 5.15 + 1.87 × 10^−3^*T* and Hv = 0.78*T* − 51.68, respectively. All sintered samples exhibit a typical alternating lamellar microstructure, while the lamellar structure of the 660 K sintered sample is significantly finer and more uniform than that of the samples sintered at lower temperatures.

The corrosion potential E_corr_ increases progressively with the elevation of sintering temperature, indicating a continuous improvement in the thermodynamic corrosion stability of the composites. All the measured corrosion current density i_corr_ values are on the order of 10^−7^ A/cm^2^ and show a monotonic downward trend as the sintering temperature rises, which directly confirms the gradual enhancement of the general corrosion resistance. Compared with the 580 K sintered sample, the 660 K sintered sample delivers a higher passivation potential E_p_, a 2.6 times wider passivation region, and a lower passivation current density i_p_. These three parameters collectively demonstrate that the passive film formed on the 660 K sintered sample can be more stable during anodic polarization and possesses significantly better structural integrity and protective performance. Moreover, the pitting potential E_pit_ of the 660 K sintered sample is also higher than that of the 580 K group, corresponding to a distinctly lower metastable pitting tendency in the 3.5 wt.% NaCl solution.

The water contact angle shows a consistent upward trend with the rise of sintering temperature for both the as-sintered polished surfaces and the corroded surfaces. However, distinct divergence in wettability variation after corrosion can be observed between the low-temperature and high-temperature sintered groups. For the samples sintered at 450 K and 510 K, the water contact angle of the corroded surface is lower than that of its corresponding as-sintered polished counterpart. In sharp contrast, for the 580 K and 660 K sintered samples, the water contact angle increases after the corrosion test, compared with their pre-corrosion polished surfaces.

## Figures and Tables

**Table 1:** Alloy composition of ball-milled powders and Cu_70_Zr_15_Ti_15_@Cu powders.

Alloy Composition (at.%)	Cu	Zr	Ti
Ball-milled powders	67.3	16.2	16.5
Cu_70_Zr_15_Ti_15_@Cu powders	85.4	7.9	6.7

**Table 2:** Amorphous fraction, Cu crystallite size, density and Vickers microhardness of Cu_70_Zr_15_Ti_15_@Cu powders and corresponding bulk samples under different sintering temperatures.

Sintering Temperature (K)	Amorphous Fraction (vol.%)	Cu Size (nm)	Density (g/cm^3^)	Hardness (Hv)
Cu_70_Zr_15_Ti_15_@Cu powders	54.8	16.1	/	/
450	30.5	18.9	6.0	297.4
510	8.3	19.5	6.1	347.7
580	0	20.2	6.2	398.5
660	0	20.7	6.4	462.2

**Table 3:** The open circuit potential (*E*_OCP_), corrosion potential (*E*_corr_), corrosion current density (*i*_corr_), passivation potential (*E*_p_), passivation current density (*i*_p_), pitting potential (*E*_pit_) of sintered bulk samples.

Temperature (K)	450	510	580	660
E_OCP_ (V)	−0.405	−0.383	−0.315	−0.246
E_corr_ (V_SCE_)	−0.375	−0.363	−0.314	−0.282
i_corr_ (×10^−7^ A/cm^2^)	8.54	7.08	1.83	1.47
E_p_ (V)	/	/	−0.279	−0.238
i_p_ (A/cm^2^)	/	/	6.59 × 10^−6^	5.69 × 10^−6^
E_pit_ (V)	/	/	−0.257	−0.180

**Table 4:** The water contact angles of as-sintered and corroded surface of the bulk samples.

Temperature (K)	450	510	580	660
As-sintered	77.4°	81.8°	89.3°	104.2°
Corroded	75.7°	78.6°	104.6°	111.9°

**Table 5:** Surface chemical composition of corroded Cu_70_Zr_15_Ti_15_@Cu bulk samples by EDS.

Temperature (K)	Cu (at.%)	Zr (at.%)	Ti (at.%)	O (at.%)	Cl (at.%)
450	31.9	7.6	4.8	52.1	3.6
510	28.1	8.2	8.3	52.5	3.0
580	26.3	8.9	9.2	53.4	2.2
660	19.0	10.0	10.9	59.5	0.7

**Table 6:** Surface chemical composition of corroded Cu_70_Zr_15_Ti_15_@Cu bulk samples by XPS.

Temperature (K)	Cu (at.%)	Zr (at.%)	Ti (at.%)	O (at.%)	Cl (at.%)	C (at.%)
450	10.8	1.8	1.4	32.0	7.2	46.8
510	7.6	2.6	2.6	32.1	4.7	50.4
580	4.0	4.1	3.2	32.6	3.4	52.8
660	2.8	4.7	3.6	33.0	1.4	54.5

**Table 7:** Equivalent circuit parameters obtained from EIS spectra of bulk samples sintered under different temperatures.

Temperature (K)	450	510	580	660
R_s_ (Ωcm^2^)	13.41	14.81	14.38	13.33
Q (10^−4^Ω^−1^cm^−2^s^n^_1_)	5.19	6.48	3.89	7.87
n_1_	0.58	0.57	0.57	0.62
R_ct_ (Ωcm^2^)	3171	3397	1048	2054
W_b_ (Ωcm^2^)	/	/	60368	93201
W_T_ (Ωs^n^_2_)	/	/	4978	8067
n_2_	/	/	0.58	0.54

## Data Availability

The original contributions presented in this study are included in the article. Further inquiries can be directed to the corresponding authors.
